# Baseline malaria burden and pyrethroid resistance in Muheza, Tanzania informing a cluster randomized trial of the 3D window screens

**DOI:** 10.1038/s41598-026-46221-6

**Published:** 2026-04-03

**Authors:** Subam Kathet, Veneranda M. Bwana, Mwantumu Ally Fereji, Jenny B. Mhando, Frank S. Magogo, Victor Mwingira, William Kisinza, Seppo Meri, Ayman Khattab

**Affiliations:** 1 Translational Immunology Research Program, Department of Bacteriology and Immunology, Faculty of Medicine, University of Helsinki, Biomedicum 1, Haartmaninkatu 8, 00014 Helsinki, Finland; 2 Amani Medical Research Centre, National Institute for Medical Research, Muheza, Tanzania; 3 HUSLAB Diagnostic Center, Helsinki University Central Hospital, 00029 Helsinki, Finland; 4 Department of Nucleic Acid Research, Genetic Engineering and Biotechnology Research Institute, City of Scientific Research and Technological Applications, New Borg El-Arab, Alexandria 21934 Egypt

**Keywords:** Baseline survey, Malaria prevalence, Vector density, Malaria risk factors, Tanzania, Insecticide resistance, Anopheles mosquitoes, Entomological inoculation rate, Diseases, Medical research, Microbiology

## Abstract

Insecticide resistance in malaria vectors has created an urgent need for alternative, non-insecticidal control tools. In response, a novel window screen, the Three-dimensional (3D) Screen, was developed. Before launching a cluster-randomized controlled trial to evaluate its impact, a baseline study was conducted in 20 hamlets (clusters) from 17 villages in Muheza District, northeastern Tanzania, to assess malaria burden, vector characteristics, and risk factors for infection. Structured questionnaires collected household information; malaria prevalence was measured among 778 children aged 6 months to 14 years using malaria rapid diagnostic tests (mRDTs), and haemoglobin was assessed by HemoCue. Indoor Centers for Disease Control and Prevention (CDC) light traps (LTs) were used for mosquito collections, yielding 14,263 mosquitoes morphologically identified, including 889 *Anopheles gambiae* sensu lato (s.l.) and 2,013 *An. funestus* senso lato (s.l.); 2,508 female *Anopheles* were further analysed by Polymerase chain reaction (PCR). World Health Organization (WHO) cylinder bioassays assessed pyrethroid resistance in *An. gambiae* s.l., and genotyping targeted the knockdown resistance (kdr)-East (L1014S) mutation. Malaria prevalence was 40.2%, and anaemia affected 55.2% of children. *An. funestus* s.l. (70.2%) and *An. gambiae* s.l. (29.8%) comprised the vector population. Mean daily entomological inoculation rates (EIRs) were 0.039 and 0.037 infectious bites per person per night, corresponding to annual EIRs of approximately 14.2 for *An. funestus* s.l., 13.5 for *An. gambiae* s.l., and 27.7 combined. Pyrethroid resistance was high, with 24-hour mortality of 56.5% to permethrin and 52.8% to deltamethrin; the kdr-East (L1014S) allele frequency in *An. gambiae s.s.* was 0.45. Multivariate analysis showed that children aged ≥ 5 years had approximately threefold higher odds of *Plasmodium* infection than those < 5 years (aOR = 3.03, 95% CI: 2.09–4.40), while sleeping under a bed net was protective (aOR = 0.36, 95% CI: 0.18–0.70). Cluster-level variance accounted for 19.5% of infection risk, highlighting community-level factors. Persistent transmission, high anaemia prevalence, and widespread insecticide resistance underscore the limitations of current measures and the need for integrated interventions, including novel non-insecticidal tools such as the 3D-Screen.

**Supplementary Information:**

The online version contains supplementary material available at 10.1038/s41598-026-46221-6.

## Introduction

Over the past two decades, significant reductions in global malaria cases and fatalities have been achieved, primarily due to malaria control initiatives focusing on the widespread use of insecticides through Long-Lasting Insecticide-treated Nets (LLINs) and Indoor Residual Sprays (IRS), along with improved diagnostic testing and prompt, efficient treatment of malaria cases^1,2^. Despite these efforts, recent years have seen a substantial increase in global malaria cases, largely due to the emergence of insecticide-resistant malaria vectors, challenges in accessing insecticide-treated bed nets, and suboptimal housing conditions, among other factors^3,4^. Furthermore, the disruption of ongoing malaria control efforts and the slow recovery of global health systems, prompted by the coronavirus disease 2019 (COVID-19) pandemic, drug resistance, humanitarian crises, and climate change, have intensified these adverse trends. According to the latest World Malaria Report by the WHO^2^, there were an estimated 282 million malaria cases worldwide in 2024, an increase of 9 million from the previous year. The African Region continues to bear the highest burden, accounting for approximately 94% of cases (about 265 million) and 95% of deaths (around 579,000). Globally, malaria claimed 610,000 lives in 2024, with children under five accounting for approximately 76% of those deaths in the African Region. More than half of all malaria deaths occurred in just four countries: Nigeria (30.3%), the Democratic Republic of Congo (11.1%), Niger (5.8%), and Tanzania (4.3%). This underscores the significant challenge malaria poses in the African Region, despite ongoing efforts to control and eliminate the disease through strategies like insecticide-treated nets (ITNs) and IRS.

Vector control is central to malaria control strategies in Africa^5,6^. Current vector control methods rely heavily on a limited number of insecticides, primarily delivered through ITNs and IRS, aimed at reducing exposure to malaria- transmitting mosquitoes. While the historical effectiveness of insecticide-based interventions is acknowledged^1,7^, the increasing prevalence of mosquito populations resistant to these chemicals has necessitated the exploration of sustainable and environmentally friendly non-insecticidal alternatives. To address this challenge, an innovative, insecticide-free intervention was developed in Finland, focusing on reducing mosquito-human contact through the use of novel window screening technology. These three-dimensional screens (3D-Screens) harness mosquitoes’ innate attraction to human hosts without relying on chemical agents. When configured as a double-layered window installation, referred to as the 3D-Window Double Screen (3D-WDS), the system functions unidirectionally, allowing mosquitoes to enter from the outside but trapping them between the two screens. In wind tunnel experiments^8^, the cone-based prototype captured 92% of mosquitoes, and subsequent semi-field trials in Tanzania^9^ demonstrated that the 3D-WDS could capture over 50% of malaria-transmitting mosquitoes.

In preparation for the next phase of evaluation, a cluster-randomised controlled trial (cRCT) was conducted in Muheza District, northeastern Tanzania, between 2020 and 2021 to assess the efficacy of the 3D-WDS in a community setting^10^. The primary objective of the trial was to determine whether the 3D-WDS, when used in conjunction with the existing standard of universal insecticide-treated net (ITN) coverage, defined as one net per two individuals, could provide additional protection against malaria transmission.

To ensure rigorous trial implementation and appropriate selection and randomisation of study clusters, it was necessary to gather comprehensive baseline data on the sociodemographic characteristics of the study area, malaria prevalence, vector bionomics, and insecticide susceptibility. Accordingly, a cross-sectional entomological and epidemiological survey was conducted, with the primary aim of mapping the district and identifying eligible hamlets for inclusion. A total of 20 hamlets (study clusters), located within 17 villages, were identified and profiled. These profiles included assessments of demographic structure, malaria prevalence, vector densities, and insecticide resistance levels. Based on these indicators, 14 hamlets were selected and randomly allocated to either the intervention arm (ITNs plus 3D-WDS) or the control arm (ITNs alone).

Muheza District in north-eastern Tanzania is an established site for malaria research and routine vector surveillance, providing an appropriate setting to characterise local vector populations, insecticide resistance, and malaria transmission patterns. The insights gained from these observational studies can be utilized for sample size calculations and site preparation for future interventional studies, contributing to the development of more effective approaches to evaluate vector control interventions in the region. Moreover, the findings from this survey played a crucial role as initial data for determining the sample size and statistical power needed for the cRCT of 3D-WDS in the chosen communities. Additionally, the demographic survey marked the initiation of district-wide project activities, serving as the first step in establishing contact and engagement with the community. This initial engagement proved beneficial before the commencement of the cRCT, allowing the investigative team to present themselves to the community and introduce the novel intervention.

This manuscript presents the findings from the baseline survey conducted in 20 selected hamlets across Muheza District. It offers a detailed account of sociodemographic conditions, malaria burden, and a comprehensive entomological profile, including vector species composition, biting rates, *Plasmodium* infection rates, the entomological inoculation rate (EIR), and insecticide resistance patterns in local *Anopheles* populations. These data provide essential context for interpreting the outcomes of the subsequent trial and for informing future vector control strategies in the region.

## Results

### Study villages

**Fig. 1:**
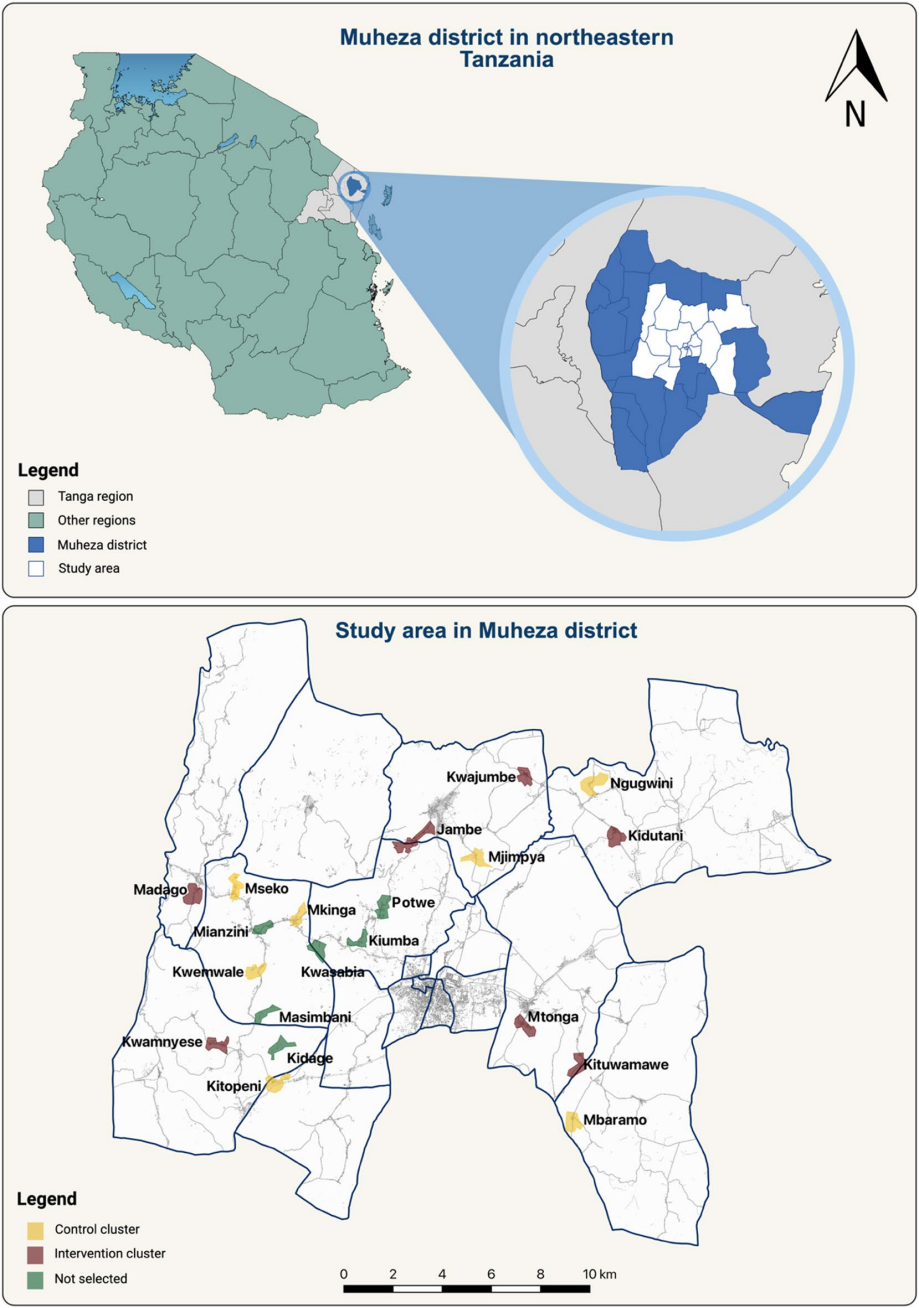
Map of the study area in Muheza District, Tanga Region, northeastern Tanzania, showing the 20 baseline candidate clusters selected for the cross-sectional survey. Intervention clusters are shown in yellow, control clusters in red, and non-selected clusters in green. The map was generated using QGIS (version 3.34, https://qgis.org) and is adapted from Kisinza et al.^10^ licensed under CC BY 4.0.”

A total of 20 hamlets (cluster hereafter) (Fig. 1) were selected from 17 villages for the baseline survey. The average cluster size was 60.2 households (95% CI: 50.6–69.7; SD ± 20.3). Clusters were chosen to ensure a minimum distance of 2 km between sites and based on factors such as household size, logistical ease, and willingness to participate. Additionally, clusters involved in other malaria intervention studies at the time of the cross-sectional survey were excluded.

### Socio-demographic profile, household characteristics and malaria prevention practices

Community sensitization, household enumeration, and data collection were conducted between May and July 2019. A total of 1,203 households fully participated in the baseline survey. The average household size was 4 persons (95% CI: 3.9–4.1), with 14.9% (*n* = 179) being single-resident households. The majority of household heads were female (64.4%), with a median age of 50 years. Housing was predominantly of traditional construction, with mud-walled houses being the most common type (62.3%). Concrete houses were the least common (12.1%). Houses were categorized into six construction types (Table 1). Most household heads (90.0%) had completed primary school. Subsistence farming was the main source of livelihood for 91.5% of households, while 8.5% were engaged in business, technical work, or services. The median reported monthly income was TSH 150,000 (≈ 60 USD). Regarding malaria prevention, 81.8% of households owned at least one LLIN. However, only 54.5% of households met the criterion for universal coverage (≥ 1 LLIN per two individuals). When assessed at the population level, 45.3% of all individuals lived in households that met universal coverage. Use of additional mosquito-control methods was reported by 36.8% of participants, mostly insecticide sprays or coils (33.5%), followed by repellents (2.5%) and burning leaves or herbs (0.8%). The majority (63.2%) reported no additional mosquito-control practices (Table 1).

**Table 1:** Demographic and social characteristics, household attributes, and malaria prevention practices in study communities of Muheza District.

Household characteristics (*N* = 1203)	Frequency	Percentage
**Household size**	4 (3.9–4.1) ± 2.3^a^	4 [2.5]^b^
Single resident households	179	14.88
2 to 3	359	29.84
4 to 6	513	42.64
> 7	152	12.64
**Number of children per household**	2 (1.9–2.1) ± 1.9^a^	2 [0.3]^b^
None	319	26.52
1 to 2	457	37.99
3 to 5	387	32.17
> 6	40	3.33
**Sex of head of household**		
Male	428	35.58
Female	775	64.42
**Age of head of household**	52(51–53) ± 17^a^	50 [38.65]^b^
15 to 25	71	5.90
26–40	298	24.77
41–65	562	46.72
> 65	272	22.61
**Education of head of household**		
No schooling	65	5.40
Primary	1083	90.02
Secondary	49	4.07
Upper Secondary	6	0.50
**Economic activity of head of household**		
Agriculture and Farming	1101	91.52
Business	51	4.24
Technician	35	2.91
Service	13	1.08
Local Healer	3	0.25
**Housing characteristics**		
**Structure**		
Burnt brick with iron sheet roof	269	22.36
Burnt brick with thatched roof	40	3.33
Mud wall with iron sheet roof	415	34.50
Mud wall with thatched roof	334	27.76
Concrete wall with iron sheet roof	102	8.48
Concrete wall with thatched roof	43	3.57
**Eaves**		
Open	1067	88.69
Closed	136	11.31
**Number of windows**	4.1 (4–4.3) ± 2.6^a^	4 [2.6]^b^
None	28	2.33
1 to 2	386	32.09
3 to 4	323	26.85
> 5	466	38.74
**Number of sleeping spaces**	2.4 (2.3–2.4) ± 1.1^a^	2 [2.3]^b^
1 to 2	700	58.19
3 to 4	457	37.99
> 5	46	3.82
**Curtain on windows**		
Yes	830	68.99
No	373	31.01
**Cooking inside house**		
Yes	505	41.98
No	698	58.02
**Wealth status (PCA-derived)**		
**Wealth quintile**		
Q1 (lowest)	243	20.2%
Q2	239	19.9%
Q3	244	20.3%
Q4	237	19.7%
Q5 (highest)	240	20.0%
**Livestock present**		
Yes	349	29.01
No	854	70.99
Malaria prevention	1.7 (1.6–1.8) ± 1.3^a^	2 [1.3]^b^
**Number of LLINs per household**		
0	219	18.20
1	338	28.10
2	340	28.26
3	235	19.53
> 3 nets	71	5.90
**Households with universal coverage of LLINs (1 LLINs per 2 individual)**		
Yes	656	54.53
No	547	45.47
**Individuals (N = 4813)**		
Individuals living in households with universal net coverage	2179	45.27
Individuals living in households without universal net coverage	2634	54.73
**Mosquito control other than nets**		
None	760	63.18
Burn leaves and herbs	10	0.83
Mosquito insecticides/coils/sprays	403	33.50
Repellents and ointments	30	2.49

^a^ Mean (95% CI) ± SD.

^b^ Median [Inter quartile range].

### Prevalence of *Plasmodium* infection, anaemia, and associated risk factors

A total of 778 children from 662 households participated in the baseline survey, drawn from 1,495 children aged 6 months to 14 years recorded during the demographic survey. The mean age of participants was 6.08 years (95% CI: 5.80–6.37; SD ± 4.02). Malaria prevalence and haemoglobin levels by cluster and age group are summarized in Table 2.

**Fig. 2:**
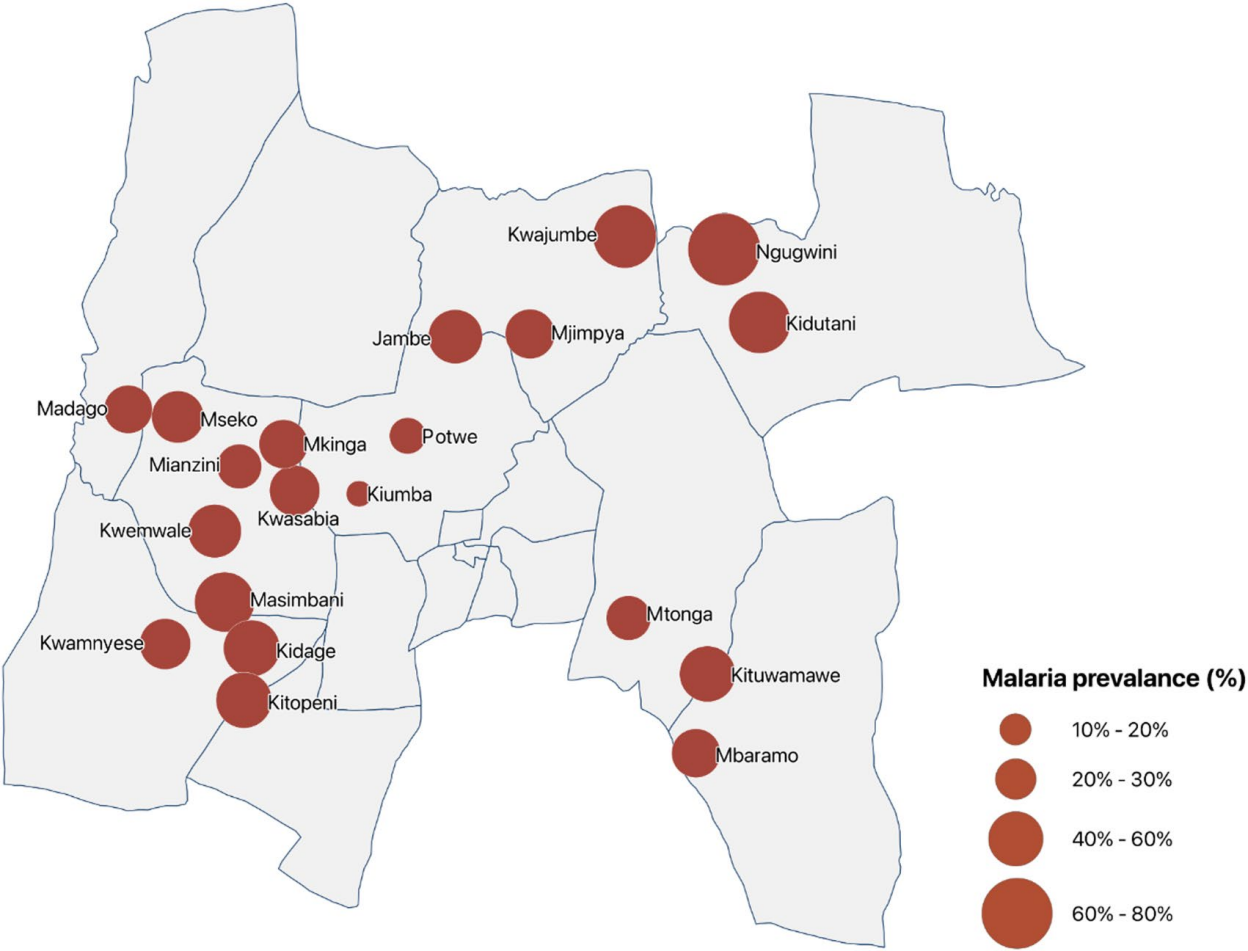
Spatial distribution of cluster-level malaria prevalence in Muheza District, Tanga Region, northeastern Tanzania. Circles represent study clusters, with bubble size proportional to malaria prevalence measured during the baseline cross-sectional survey. The map was generated using QGIS (version 3.34, https://qgis.org).

The overall prevalence of Plasmodium infection by mRDT was 40.2% (95% CI: 36.8–43.7). Clinical malaria prevalence, defined as an axillary temperature ≥ 37.5 °C plus a positive mRDT, was 5.3% (95% CI: 2.9–7.6). Infection prevalence varied widely across clusters, with the highest rates in Ngugwini (78.8%) and Kwajumbe (59.5%), and the lowest in Kiumba (9.1%) and Potwe (19.1%) (Fig. 2).

Mean haemoglobin (Hb) concentrations (g/dL) among children aged 6–59 months, 5–11 years, and 11–14 years were 10.65 (95% CI: 5.40–14.20), 11.33 (95% CI: 6.00–15.50), and 11.65 (95% CI: 8.60–14.40), respectively. The overall mean Hb concentration was 11.10 g/dL (95% CI: 5.40–15.50). More than half of participants (55.2%, 426/778; 95% CI: 48.8–61.5) had mild to severe anaemia, and 50.9% (217/426) of anaemic children tested positive for Plasmodium.

Children with Plasmodium infection had approximately three times the odds of moderate-to-severe anaemia compared with uninfected children (OR = 3.01, 95% CI: 2.15–4.26; *p* < 0.001). Definitions of all variables included in subsequent risk factor analyses are provided in Table 3.

In the univariate analysis, several factors showed associations with Plasmodium infection at *p* < 0.20 (Table 4). Children aged ≥ 5 years had significantly higher odds of infection compared with those < 5 years (OR = 3.07, 95% CI: 2.16–4.39; *p* < 0.001). Sleeping under a bed net the previous night was protective (OR = 0.41, 95% CI: 0.24–0.69; *p* = 0.001).

Two housing-related variables demonstrated non-significant but notable trends: children in households with more than six windows had lower odds of infection compared with those with fewer than three (OR = 0.68, 95% CI: 0.39–1.15; *p* = 0.151), while open eaves were associated with higher odds of infection compared with closed eaves (OR = 1.59, 95% CI: 0.86–2.99; *p* = 0.140).

Wealth quintiles showed a graded but non-significant decrease in infection risk, with the richest households exhibiting the lowest infection prevalence (Q5 vs. Q1: OR = 0.70, 95% CI: 0.44–1.10; *p* = 0.122). Full univariate results, including all demographic, socioeconomic and housing variables, are provided in Supplementary Table S1.

In the multilevel logistic regression model, which included a random intercept to account for clustering at the hamlet level, only child age and bed-net use remained independently associated with Plasmodium infection (Table 5). Children aged ≥ 5 years had 2.78 times higher odds of infection compared with younger children (aOR = 2.78, 95% CI: 1.99–3.89; *p* < 0.001). Sleeping under a bed net remained strongly protective (aOR = 0.34, 95% CI: 0.19–0.62; *p* < 0.001).

No other demographic, socioeconomic or housing characteristics were independently associated with infection after adjustment, including child sex, household head age, household head sex, household size, household head education, wealth quintile, or household ITN access (all *p* > 0.05). As per the predefined modelling strategy, all mandatory covariates were retained in the multilevel model regardless of statistical significance; however, none of these variables demonstrated an independent association with Plasmodium infection after adjustment.

The Intraclass Correlation Coefficient (ICC) at the cluster level was 0.195, indicating substantial clustering of infections within hamlets (Table 5).

**Table 2:** Prevalence of malaria (RDT) and mean haemoglobin concentrations (g/dl) among children aged 6 months to 14 years in the study clusters of Muheza District, Tanzania, June–August 2019.

SN	Study cluster	Total HH	Total population	Total population (6 M - 14 Y)	RDT Results			Mean hemoglobin concentrations (g/dl) (95% CI of Mean)			
					*N* (Total)	Positive. *n* (%)	95% CI for positives	Total participants	6–59 months	5–11 years	12–14 years
1	Mbaramo	68	274	76	45	16 (35.6)	(23.2–50.2)	11.57 (7.40–14.80)	10.81 (7.40–13.20)	11.91 (10.10–14.80)	12.12 (9.60–13.80)
2	Kituwamawe	44	173	52	28	13 (34.2)	(29.5–64.2)	11.05 (7.40–14.40)	10.17 (7.40–12.70)	11.32 (9.20–12.80)	12.8 (11.20–14.40)
3	Mtonga	43	214	77	40	12 (30.0)	(18.1–45.4)	10.96 (8.00-13.50)	10.4 (8.00–12.00)	11.16 (8.50–12.90)	11.71 (10.20–13.50)
4	Kidutani	53	210	75	42	24 (57.1)	(42.2–70.9)	10.48 (5.40–13.20)	10.2 (5.40–12.70)	10.82 (7.30–13.20)	**
5	Ngugwini	62	204	58	33	26 (78.8)	(62.2–89.3)	10.45 (6.80–12.50)	10.26 (6.80–12.50)	10.5 (8.10–11.90)	10.65 (8.60–11.90)
6	Kwajumbe	67	246	87	42	25 (59.5)	(44.5–73.0)	10.43 (6.00-12.50)	10.43 (7.50–12.50)	10.4 (6.00-12.40)	10.8 (10.40–11.20)
7	Mjimpya	65	271	91	41	15 (36.6)	(23.6–51.9)	11.28 (8.50–14.20)	11.35 (9.90–14.20)	11.28 (8.50–12.80)	11.07 (10.30–12.00)
8	Jambe	80	361	88	46	20 (43.5)	(30.2–57.8)	10.55 (6.80–13.60)	10 (6.80–11.90)	11.19 (9.50–13.60)	11.48 (9.70–12.70)
9	Potwe	107	423	131	47	9 (19.1)	(10.4–32.5)	11.43 (6.40–14.20)	10.83 (6.40–13.20)	12.05 (9.90–14.20)	12.1 (8.90–13.30)
10	Kiumba	69	259	87	44	4 (9.1)	(3.6–21.2)	11.92 (9.60–15.50)	11.28 (9.60–13.30)	12.44 (10.00-15.50)	12.06 (11.40–12.50)
11	Kwasabia	93	352	88	45	17 (37.8)	(25.1–52.4)	11.45 (8.90–13.90)	10.69 (8.90–12.40)	11.72 (9.60–13.10)	12.23 (10.80–13.90)
12	Mkinga	49	230	86	45	16 (35.6)	(23.2–50.2)	11.03 (7.20–14.00)	10.71 (7.20–12.20)	11.12 (7.80–12.60)	11.86 (11.00–14.00)
13	Mianzini	54	218	61	34	10 (29.4)	(16.8–46.2)	11.6 (8.10–13.30)	11.36 (8.10–13.30)	11.96 (10.20–13.30)	10.74 (9.80–13.30)
14	Mseko	42	188	58	32	13 (40.6)	(25.5–57.7)	10.82 (8.20–12.60)	11.13 (9.70–12.10)	10.55 (8.20–12.50)	10.78 (9.20–12.60)
15	Madago	61	225	73	41	14 (34.1)	(21.6–49.5)	11.13 (9.30–13.10)	10.94 (9.90–13.00)	11.34 (9.30–13.10)	10.93 (9.60–11.70)
16	Kwemwale	52	201	66	35	15 (42.9)	(28.0–59.1)	10.67 (7.10–14.10)	10.56 (8.40–14.10)	10.6 (7.10–13.00)	11.22 (10.40–12.00)
17	Masimbani	25	105	39	26	14 (53.4)	(35.5–71.2)	11.41 (9.60–14.00)	11.14 (9.60–12.80)	11.3 (9.60–13.60)	11.93 (10.20–14.00)
18	Kwamnyese	49	163	53	31	12 (38.7)	(23.7–56.2)	11.28 (8.10–13.90)	10.77 (9.20–12.70)	11.61 (8.10–13.90)	12.02 (9.90–13.80)
19	Kidage	33	145	40	23	11 (47.8)	(29.2–67.0)	10.9 (8.70–13.40)	10.7 (8.70–12.00)	10.85 (9.30–13.10)	12.4 (11.40–13.40)
20	kitopeni	87	351	105	58	27 (46.6)	(34.3–59.2)	11.3 (6.10–14.40)	10.5 (6.10–13.70)	11.47 (7.40–14.40)	11.76 (9.60–13.30)
Total		**1**,**203**	**4**,**813**	**1**,**491**	**778**	**313 (40.2)**	**(36.8–43.7)**	**11.1 (5.40–15.50)**	**10.65 (5.40–14.20)**	**11.33 (6.00-15.50)**	**11.65 (8.60–14.40)**

**Table 3:** Definitions of variables used in malaria risk factor analysis.

SN	Variables	Description
1	HH head age group	Age was categorized as binary variable where ‘above mean’ means above average age and ‘below mean’ represents below the average age
2	HH hed gender	Binary variable ‘Male’ and ‘Female’
3	HH head went to school?	Binary variable ‘yes’ if the head of household has at least completed primary education
4	Economic activity	Binary ' Agriculture’ and ‘Others’
5	HH Size	Household size was categorized in three groups based in total individuals living in the household, 2–4 individuals, 5–7 individuals and > 7 individuals
6	Improved housing	Is a binary variable where houses are coded as ‘yes’ if they have at least one of the following, intact ceiling, brick walls, plastered walls, full cement floors or iron roofs
7	Number of sleeping spaces	Number of sleeping space was categorized into three groups based on total rooms available for sleeping, 1–2 spaces, 3–4 spaces and > 5 spaces.
8	ITN ownership	Binary variable ‘yes’ if household owns at least one ITN’s
9	Window present	Binary variable ‘yes’ if house is built with windows
10	Number of windows	Categorized into three groups Windows less than 3, 3–5, more than 5
11	Curtain present	Binary variable ‘yes’ if household has curtains on windows
12	Eave type	Binary variable ‘open’ if household has open eaves or ‘closed’ if household has no eaves
13	Cooking inside the house	Binary variable ‘yes’ if cooking is done indoors
14	Using additional mosquito control method	Binary variable ‘yes’ if household uses additional control method
15	Age group (children between 6 months and 14 years of age)	Binary variable: ‘below 5 years’ or ‘above 5 years’
16	Gender (participating children)	Binary variable ‘Male’ and ‘Female’
17	Slept under the net last night?	Binary variable, ‘yes’ if the child slept under the bed net pervious night

**Table 4:** Univariate logistic regression analysis of factors associated with *Plasmodium* infection among children aged 6 months–14 years.

Variable	Number of children (*N* = 778)	Children with Plasmodium infection (%)	Odds Ratios (OR)	95% CI	*p*-value
**HH head age group**					
< Mean (Ref)	496	207 (41.7)	1		
> Mean	282	106 (37.6)	0.846	0.57–1.23	0.389
**HH head gender**					
Male (Ref)	560	222 (39.6)	1		
Female	218	91 (41.7)	1.191	0.81–1.74	0.372
**HH head went to school?**					
No (Ref)	35	15 (42.9)	1		
Yes	743	298 (40.1)	1.021	0.45–2.31	0.961
**Economic activity**					
Agriculture (Ref)	722	294 (40.7)	1		
Others	56	19 (33.9)	0.868	0.44–1.66	0.675
**HH size**					
2 to 4 (Ref)	246	97 (39.4)	1		
5 to 7	403	161 (40.0)	1.038	0.68–1.56	0.859
> 7	129	55 (42.6)	1.085	0.57–2.04	0.801
**Income**					
Below average (Ref)	491	196 (39.9)	1		
Above average	287	117 (40.8)	1.181	0.79–1.76	0.413
**Improved housing**					
No (Ref)	539	228 (42.3)	1		
Yes	239	85 (35.6)	0.690	0.30–1.58	0.379
**Number of sleeping spaces**					
1 to 2 (Ref)	383	161 (42.0)	1		
3 to 4	361	138 (38.2)	0.888	0.58–1.34	0.580
> 5	34	14 (41.2)	0.925	0.35–2.32	0.869
**ITN ownership**					
No (Ref)	112	42 (37.5)	1		
Yes	666	271 (40.7)	0.87	0.58–1.32	0.524
**Window present**					
No (Ref)	10	4 (40.0)	1		
Yes	768	309 (40.2)	1.175	0.25–6.11	0.839
**Number of windows**					
< 3 (Ref)	236	102 (43.2)	1		
3 to 5	301	125 (41.5)	0.914	0.59–1.39	0.678
> 6	241	86 (35.7)	0.677	0.39–1.15	0.151
**Curtain present**					
No (Ref)	544	228 (41.9)	1		
Yes	234	85 (36.3)	0.907	0.62–1.31	0.605
**Eave type**					
Closed (Ref)	78	24 (30.8)	1		
Open	700	289 (41.2)	1.592	0.86–2.99	0.140
**Cooking inside the house**					
No (Ref)	465	181 (38.9)	1		
Yes	313	132 (42.2)	1.097	0.76–1.57	0.615
**Using additional mosquito control method**					
No (Ref)	690	277 (40.1)	1		
Yes	88	36 (40.9)	1.042	0.60–1.77	0.880
**Age group**					
Below 5 (Ref)	314	86 (27.4)	1		
Above 5	464	227 (48.9)	3.072	2.16–4.39	< 0.001
**Gender (participated children)**					
Male (Ref)	395	166 (42.0)	1		
Female	383	147 (38.4)	1.012	0.72–1.41	0.945
**Slept under bed net last night?**					
No (Ref)	61	37 (60.7)	1		
Yes	717	276 (38.5)	0.41	0.24–0.69	0.001
**Wealth quintile**					
Q1 (Poorest, Ref)	156	72 (46.2%)	1		
Q2	156	65 (41.7%)	0.95	0.61–1.48	0.824
Q3	156	58 (37.2%)	0.74	0.47–1.16	0.185
Q4	156	54 (34.6%)	0.69	0.45–1.08	0.106
Q5 (Richest)	154	44 (28.6%)	0.70	0.44–1.10	0.122

Ref = Reference category.

## Entomological findings

### Vector species composition

**Fig. 3:**
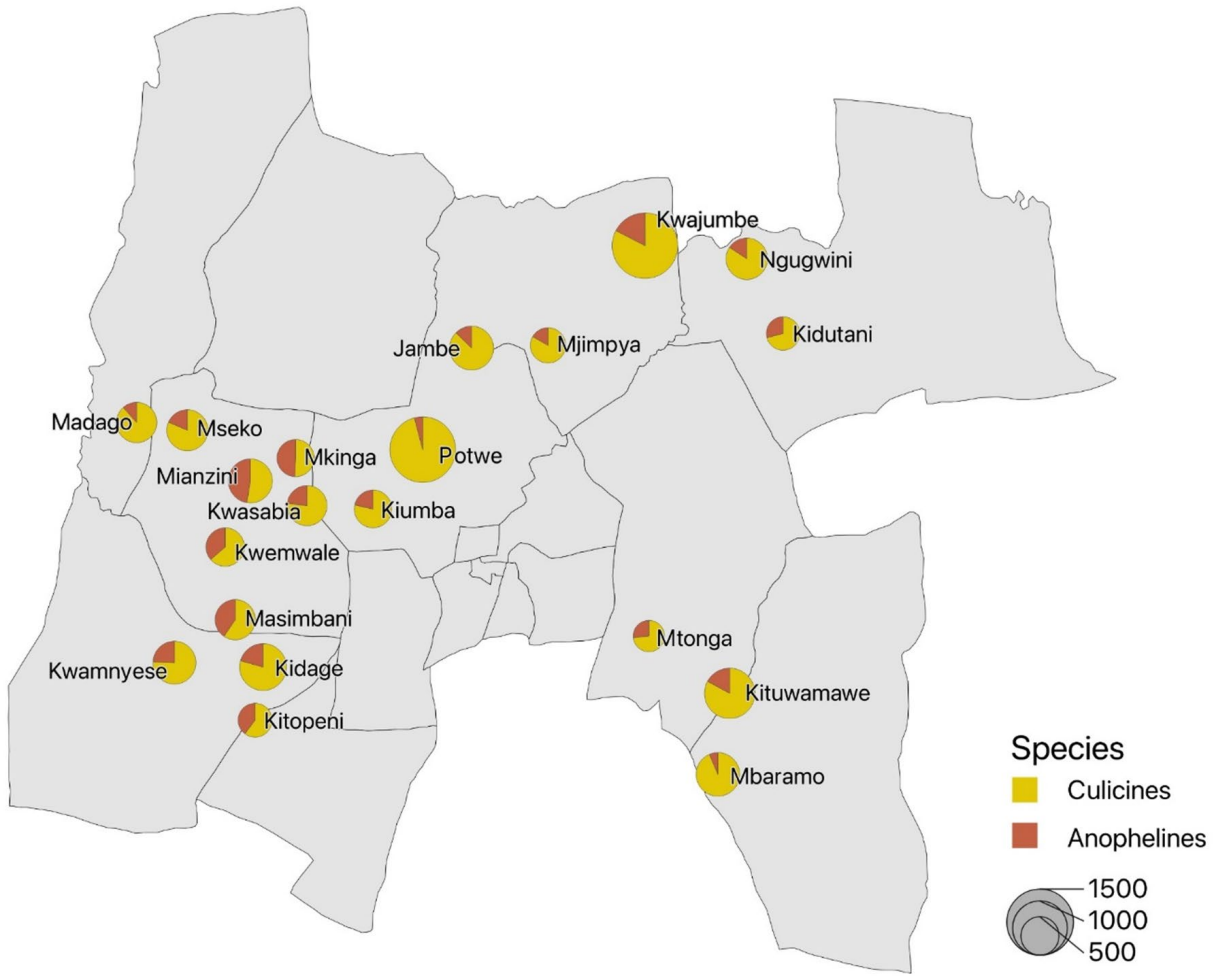
Indoor mosquito collections by species group across 20 baseline clusters in Muheza District, Tanga Region, northeastern Tanzania. Pie charts represent the relative proportions of anophelines and culicines collected indoors using CDC-LTs, with each pie corresponding to a study cluster. The map was generated using QGIS (version 3.34, https://qgis.org).

**Fig. 4:**
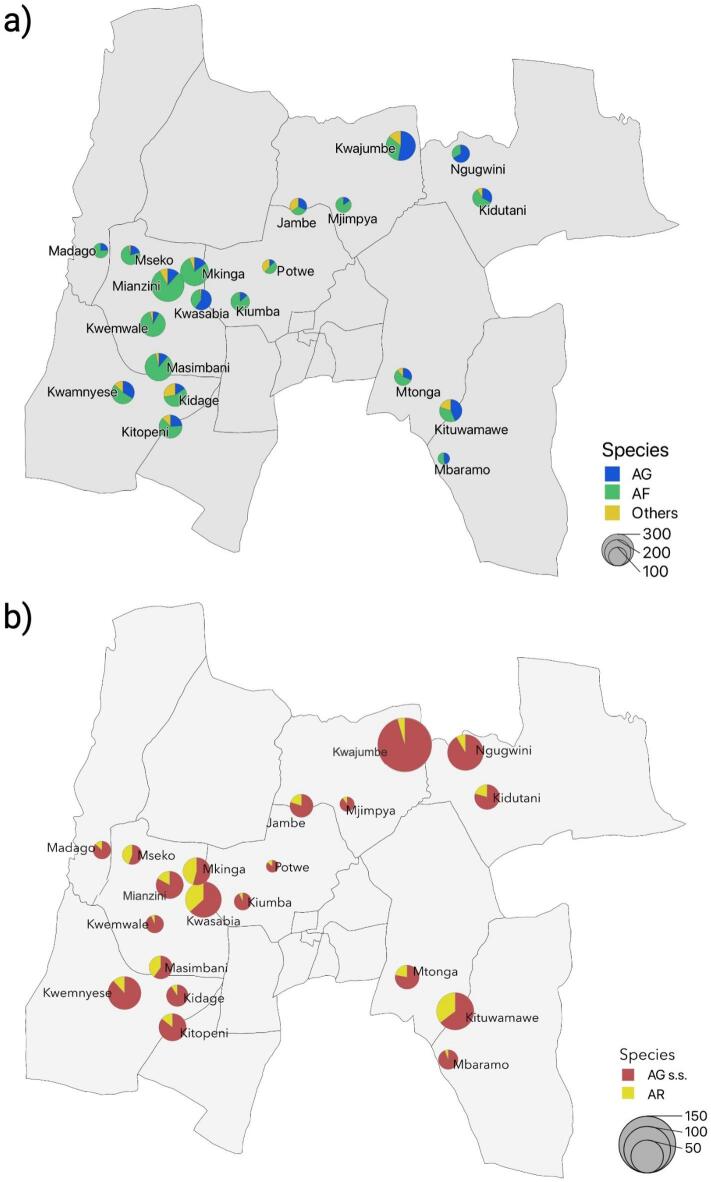
Anopheline species composition and molecular identification across study clusters in Muheza District, Tanga Region, northeastern Tanzania. (**a**) Species composition of indoor collected anophelines based on morphological identification, showing the relative proportions of *Anopheles gambiae* s.l. (AG) and *Anopheles funestus* (AF) group across clusters. (**b**) PCR-based identification of sibling species within the *Anopheles gambiae* complex, showing the relative proportions of *Anopheles gambiae* s.s. (AG s.s.) and *Anopheles arabiensis* (AR). Pie charts represent proportional species composition, and pie size reflects the total number of mosquitoes collected per cluster. Maps were generated using QGIS (version 3.34, https://qgis.org).

Indoor mosquito collection composed of both Anophelines and Culicines specimens with Culicines being the predominant species in the overall vector composition (Fig. 3). A total of 14,263 (7.9% male, *n* = 1,132, 92.1% female, *n* = 13,131) mosquitoes were captured using indoor light trapping of which 3,202 were morphologically identified as Anophelines (12.8% male, *n* = 410, 87.2% female, *n* = 2,792) and 11,061 were identified as Culicines (7.0% male, *n* = 772, 93.0% female, *n* = 10,339). Anophelines composed of 889 *An. gambiae* s.l. (16.0% male, *n* = 142, 84.0% female, *n* = 747), 2013 *An. funestus* s.l. (12.5% male, *n* = 252, 87.5% female, *n* = 1,761) and 300 specimen of unidentified Anophelines (5.3% male, *n* = 16, 94.7% female, *n* = 284) (Fig. 4a). A total of 2,508 specimen identified as female *An. gambiae* s.l. and *An. funestus* s.l. were subjected to multiplex PCR for sibling species identification. Of 747 *An. gambiae* s.l. subjected to molecular analysis, 76.2% (*n* = 569) were identified as *An. gambiae* s.s., 17.8% (*n* = 133) were identified as *An. arabiensis*, a single specimen was identified as *An. quadriannulatus* and, 5.9% (*n* = 44) specimens either failed to amplify, or a band wasn’t seen during visualization. The molecular analysis of sibling species within the *An. gambiae* complex showed that *An. gambiae* s.s. was the prevailing species in the study area (Fig. 4b). Of 1,761 female *An. funestus* s.l. subjected to molecular analysis, 94.6% (*n* = 1,666) were identified as *An. funestus* s.s., 1.0% (*n* = 17) as *An. leesoni*, 1.7% (*n* = 30) as *An. rivulorum*, a single specimen as *An. parensis* and 2.7% (*n* = 47) either failed to amplify or a band wasn’t seen during visualization concluding that *An. funestus* s.s. was the predominant vector within *An. funestus* species complex in the study area.

**Table 5:** Multivariate mixed-effects logistic regression analysis of factors associated with *Plasmodium* infection among children aged 6 months–14 years.

Variable	Comparison	Adjusted OR	95% CI	*p*-value
Age group of child	≥ 5 years vs. < 5 years (Ref)	2.78	1.99–3.89	< 0.001
Sex of child	Female vs. Male (Ref)	0.92	0.67–1.25	0.579
Slept under bed net last night	Yes vs. No (Ref)	0.34	0.19–0.62	< 0.001
Household head age	Below mean vs. Above mean (Ref)	1.22	0.86–1.73	0.264
Household head sex	Male vs. Female (Ref)	0.87	0.60–1.27	0.465
Household size	5–7 vs. 2–4 (Ref)	1.04	0.72–1.51	0.827
	≥ 8 vs. 2–4 (Ref)	1.40	0.83–2.35	0.203
Education of household head	Primary vs. None (Ref)	0.86	0.39–1.92	0.714
	Secondary vs. None (Ref)	0.71	0.20–2.56	0.605
Wealth quintile	Q2 vs. Q1 (Ref)	1.02	0.62–1.69	0.927
	Q3 vs. Q1	0.73	0.42–1.26	0.255
	Q4 vs. Q1	0.84	0.50–1.41	0.508
	Q5 vs. Q1	0.71	0.41–1.23	0.219
Household ITN access	Yes vs. No (Ref)	1.81	0.95–3.43	0.070

Ref = Reference category, ICC for village was 0.195.

### Indoor mosquito density, human biting rates and *Plasmodium* infectivity of Anophelines

The mean mosquito density was calculated as the average number of mosquitoes collected per cluster per night across the study area. The human biting rate (HBR), expressed as bites per person per night (b/p/n), was derived for each cluster and species. Detailed cluster-level results for *Anopheles gambiae* s.l. and *Anopheles funestus* s.l. are presented in supplementary Table S2.

Across all 20 study clusters, the combined average number of *Anopheles* mosquitoes (both species) collected was 125 (95% CI: 93.50–157.00), ranging from 37 in Potwe to 291 in Mianzini. The mean indoor vector density per night was 10.50 (95% CI: 7.79–13.10), with cluster-specific values ranging from 3.80 (Potwe) to 24.39 (Mianzini). The overall average HBR was 3.48 b/p/n (95% CI: 2.60–4.37), varying from 1.03 in Potwe to 8.08 in Mianzini.

For *An. gambiae* s.l., the mean total collection was 37.35 (95% CI: 23.41–51.29) per cluster, ranging from 8 (Potwe) to 136 (Kwajumbe). The mean indoor density was 3.11 per night (95% CI: 1.95–4.27), with cluster-specific values from 0.67 (Potwe) to 11.30 (Kwajumbe) and the mean HBR was 1.04 b/p/n (95% CI: 0.65–1.43), ranging from 0.22 in Potwe to 3.78 in Kwajumbe.

For *An. funestus* s.l., the mean total collection was 88.05 (95% CI: 58.50–117.60) per cluster, ranging from 22 (Mbaramo) to 254 (Mianzini). The mean indoor density was 7.34 per night (95% CI: 4.87–9.81), with cluster-specific values between 1.83 (Mbaramo) and 21.20 (Mianzini) and, the mean HBR was 2.45 b/p/n (95% CI: 1.38–3.52), ranging from 0.61 in Mbaramo to 7.06 in Mianzini.

PCR analysis was conducted on 2,508 specimens, including both female *An. gambiae* s.l. and *An. funestus* s.l., revealing an overall *Plasmodium* infection rate of 2.2% (*n* = 54/2,508). In *An. gambiae* s.l., the mean infection rate across the study area was 3.0% (CI: 1.5% − 4.5%), with an overall infection rate of 3.5% (*n* = 26/747). Within the *An. gambiae* complex, 3.7% (*n* = 21/569) of *An. gambiae* s.s. and 3.8% (*n* = 5/133) of *An. arabiensis* were infected with *Plasmodium*. The overall mean infection rate for *An. gambiae* s.s. and *An. arabiensis* across the study area was 3.6% (CI: 1.6% − 5.7%) and 3.2% (CI: -0.8% − 7.2%), respectively. Subsequent PCR analysis of *An. funestus* s.l. specimens revealed an overall infection rate of 1.6% (*n* = 28/1,761), with the mean infection rate across the study area being 2.1% (CI: 0.5% − 3.8%).

The mean daily EIR was 0.037 (95% CI: 0.014–0.060; SD ± 0.047) for *An. gambiae* s.l., 0.039 (95% CI: 0.018–0.060; SD ± 0.044) for *An. funestus* s.l., and 0.076 (95% CI: 0.046–0.104; SD ± 0.060) for all *Anopheles* combined. These correspond to annual EIRs of approximately 13.5 for *An. gambiae* s.l., 14.2 for *An. funestus* s.l., and 27.3 for all *Anopheles* combined. The coefficient of variation was highest for *An. gambiae* s.l. (129.9%), followed by *An. funestus* s.l. (111.9%) and all *Anopheles* combined (82.9%), indicating greater relative dispersion in *An. gambiae* s.l. transmission. A fully adjusted Negative Binomial regression model (offset by log trap-nights) indicated that none of the household-, socio-demographic-, or housing-structure characteristics were significantly associated with cluster-level EIR. This suggests that the marked inter-cluster heterogeneity in mosquito density, HBR and infection rates described above was not explained by the measured household-level covariates (Supplementary Table S3).

In the species-specific Negative Binomial model, *An. funestus* s.l. did not differ from *An. gambiae* s.l. in adjusted species-specific EIR (RR = 0.97; 95% CI: 0.40–2.37; *p* = 0.96), indicating that both vector groups contributed similarly to baseline transmission intensity (Supplementary Table S4).

At the individual mosquito level, a cluster-adjusted Generalized Estimating Equation (GEE) logistic regression showed that *An. funestus* s.l. had lower odds of *Plasmodium* infection compared with *An. gambiae* s.l. (OR ≈ 0.50; 95% CI: 0.26–0.96; *p* = 0.038). This pattern indicates that although *An. funestus* s.l. mosquitoes were individually less likely to carry *Plasmodium*, their higher abundance in some clusters offset this difference, resulting in no detectable difference in species-specific EIR at baseline.

### *Anopheles* species and malaria transmission

The analysis of the relationship between malaria prevalence and the distribution of Anopheles species collected with indoor CDC light traps (CDC-LTs) revealed that *An. gambiae* s.l. was the primary contributor to malaria transmission in the study area. This species showed a moderate positive and statistically significant correlation with malaria prevalence (*r* = 0.5274, *p* = 0.0169), with variation in *An. gambiae* s.l. explaining approximately 27.8% of the variance in malaria prevalence during the cross-sectional survey. In contrast, *An. funestus* s.l. showed a moderate negative correlation (*r* = -0.4224), which was not statistically significant (*p* = 0.0635), indicating that its contribution to malaria prevalence could not be confirmed. The combined proportions of *An. gambiae* s.l. and *An. funestus* s.l., as well as other Anopheles species, exhibited only weak and non-significant correlations with malaria prevalence. Overall, these findings highlight *An. gambiae* s.l. as the key vector associated with malaria prevalence in the study area.

### Bloodmeal sources and host seeking behaviour

Out of 243 visibly engorged *Anopheles* specimens examined by PCR, the overall human blood index (HBI) was 0.55. *An. funestus* s.l. exhibited a notably higher HBI of 0.64 in contrast to 0.32 for *An. gambiae* s.l. (supplementary Table S5). The HBI for *An. gambiae* s.s. and *An. arabiensis* was relatively similar at 0.32 and 0.36, respectively. *An. gambiae* s.l. showed an overall bovine blood index (BBI) of 0.01, while *An. funestus* s.l. had a BBI of 0.07. Although other blood meal sources were identified, their occurrence was relatively low compared to human blood. A significant number of specimens displayed no visible band for cytochrome b gene fragment during PCR analysis. These unknown samples underwent sibling species analysis, indicating no DNA damage, suggesting that the absence of PCR bands was likely due to blood meals from unidentified sources. The overall blood meal from unknown sources was 0.34.

### Pyrethroid susceptibility

**Fig. 5:**
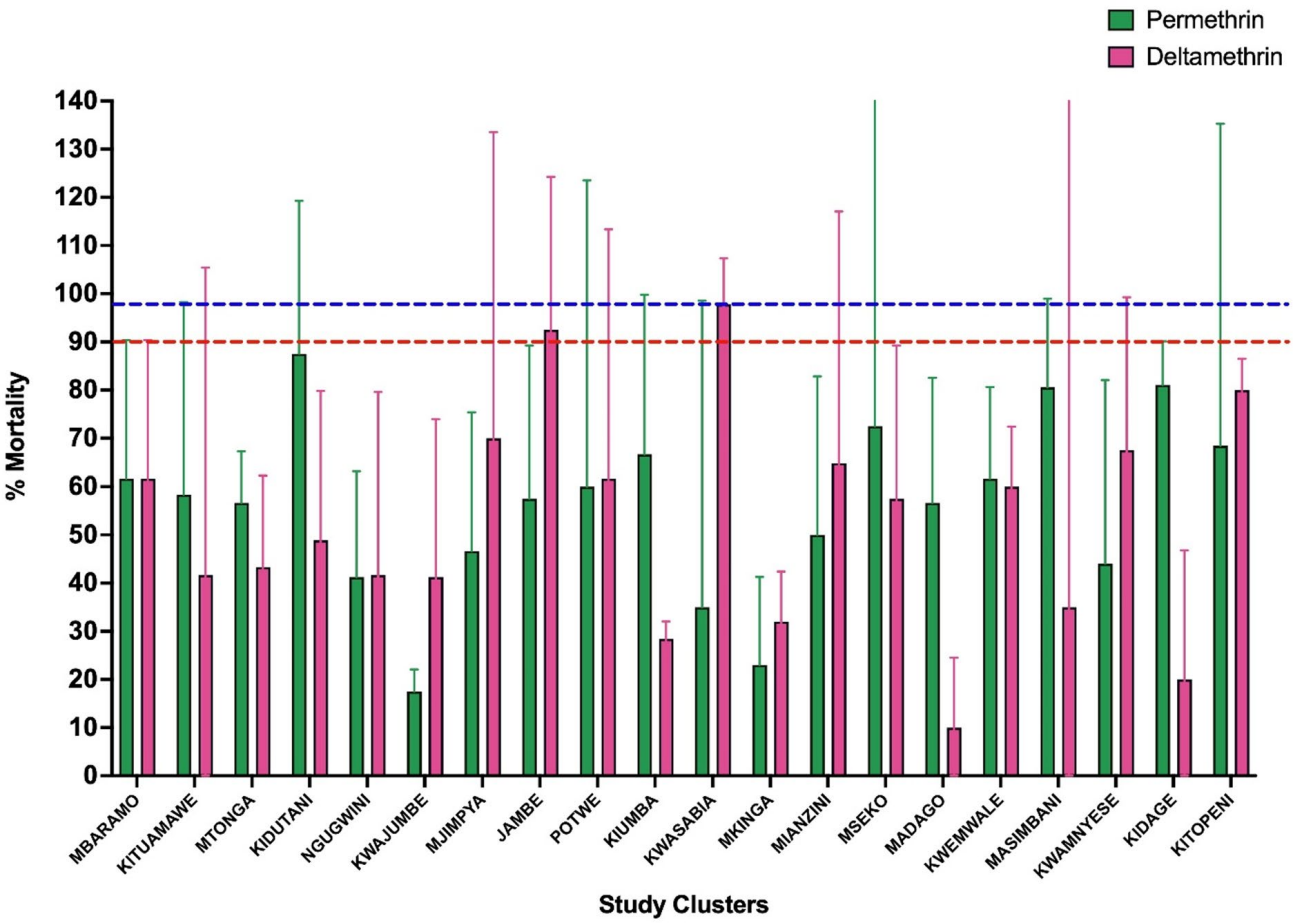
Percentage corrected mortality (CoM) of *An. gambiae* s.l. exposed to discriminating concentrations of permethrin and deltamethrin in twenty study clusters across Muheza District. Bars represent cluster means. Error bars show the 95% confidence interval calculated from replicate measurements. The red-dotted line at 90% mortality indicates possible resistance, while the blue-dotted line at 98% indicates susceptibility. Mortality below 90% indicates confirmed resistance. The figure was generated using GraphPad Prism version 10.2.2 (GraphPad Software, https://www.graphpad.com).

The *An. gambiae* s.l. population exhibited high resistance levels against the discriminating concentrations of both pyrethroid permethrin (0.75%) and deltamethrin (0.05%). The average mortality rate with the discriminating concentration of permethrin (0.75%) was 56.5% (CI: 47.9% − 65.1%), ranging from 17.5% (Kwajumbe) to 87.5% (Kidutani). For deltamethrin (0.05%), the average mortality rate was 52.8% (CI: 42.0% − 63.5%), with a range from 10.0% (Madago) to 97.9% (Kwasabia). Significant variation in insecticide resistance levels was observed across the study area against commonly used pyrethroids (Fig. 5, supplementary Table S6).

### Distribution of kdr-East (L1014S) in *An. gambiae* s.l. in Muheza district

A subset of approximately 50% of live and dead *An. gambiae* s.l. specimens (*n* = 1185) subjected to the WHO insecticide susceptibility tests were genotyped for sibling species identification and to detect the presence of the knockdown resistance-East (kdr-East) L1014S allele using a TaqMan assay. Among these specimens, 11.7% (*n* = 139) were identified as *An. gambiae s.s.* and 88.3% (*n* = 1046) as *An. arabiensis* using PCR techniques (Supplementary Table S7).

When genotyped for L1014S, 1094 specimens were homozygous for the susceptible wild type (SS), 64 were homozygous resistant (RR), and 27 were heterozygous (RS). The frequency of the L1014S kdr-East mutation in *An. gambiae* s.l. across the study area was 6.5% (95% CI: 5.6–7.6%). The allele frequency was substantially higher in *An. gambiae s.s.* (45.0%; 95% CI: 39.2–50.8%) compared with *An. arabiensis* (1.4%; 95% CI: 1.0–2.0%).

There was a significant difference in the L1014S allele frequency between resistant and susceptible *An. gambiae* s.l. (χ² = 11.76; *p* = 0.0028) and within *An. gambiae s.s.* (χ² = 14.21; *p* = 0.0008); however, no significant difference was observed between resistant and susceptible *An. arabiensis* specimens (Fisher’s exact test, *p* = 0.3842). Of the total 64 homozygous resistant *An. gambiae* s.l. identified through genotyping, 81.0% (*n* = 52) were found in *An. gambiae s.s.* specimens. Notably, 37.4% (52/139) of all *An. gambiae s.s.* were homozygous for L1014S, indicating a high level of genotypic resistance to pyrethroids within this sibling species.

The distribution of kdr-East (L1014S) mutations in *An. gambiae* s.l. in Muheza is shown in Table 6, and Supplementary Table S7 outlines the distribution of kdr-East alleles across the 20 study clusters in Muheza district.

**Table 6:** Distribution of kdr-East (L1014S) in *An. gambiae* s.l. in Muheza district.

Specimens	*N*	RR	RS	SS	*R* freq (95% CI)	Statistics
*An. gambiae s.s.*						
Resistant (surviving)	71	37	10	24	59.2% (50.9–66.9%)	
Susceptible (dead)	68	15	11	42	30.1% (23.1–38.3%)	χ² = 14.21; *p* = 0.0008
Total	139	52	21	66	45.0% (39.2–50.8%)	
*An. arabiensis*						
Resistant (surviving)	517	8	2	507	1.7% (1.1–2.7%)	
Susceptible (dead)	529	4	4	521	1.1% (0.6–2.0%)	Fisher’s exact test; *p* = 0.3842
Total	1046	12	6	1028	1.4% (1.0–2.0%)	
*An. gambiae s.l. (pooled)*						
Resistant (surviving)	588	45	12	531	8.7% (7.2–10.4%)	
Susceptible (dead)	597	19	15	563	4.4% (3.4–5.8%)	χ² = 11.76; *p* = 0.0028
Total	1185	64	27	1094	6.5% (5.6–7.6%)	

N: number of mosquitoes genotyped; SS: susceptible; RS heterozygote; RR resistant.

## Discussion

This study provides a comprehensive baseline of the sociodemographic, epidemiological, and entomological characteristics of malaria in Muheza District, northeastern Tanzania, a high burden setting. The findings highlight persistent challenges in control: high malaria prevalence, widespread pyrethroid resistance, and vector populations dominated indoors by *An. funestus* s.l., while *An. gambiae* s.l. showed the strongest association with malaria prevalence. These insights informed the design and implementation of a cRCT evaluating the 3D-WDS, a non-insecticidal intervention.

The cross-sectional survey (May–August 2019) preceded the cRCT to establish baseline transmission indicators during the long-rains peak. Conducting the survey before randomisation ensured intervention and control clusters were comparable in epidemiological and entomological profiles (malaria prevalence, mosquito densities). House mapping also quantified the proportion of dwellings suitable for 3D-WDS deployment.

Mainland Tanzania remains at substantial malaria risk with heterogeneous transmission^11–13^. In our study, *Plasmodium* infection prevalence among children 6 months–14 years was 40.2%, with 5.3% meeting the case definition for clinical malaria, consistent with persistent transmission in northeastern Tanzania^14,15^. Prevalence varied markedly by cluster (9.1%–78.8%), suggesting localised differences in vector density, housing characteristics, and control practices.

Children aged ≥ 5 years had higher infection odds than those < 5 years (aOR = 3.03, 95% CI: 2.09–4.40), likely reflecting greater exposure (time outdoors, less consistent net use). Infection was strongly associated with moderate-to-severe anaemia, underscoring malaria’s impact on child health and aligning with prior evidence from endemic settings^16^.

Bed-net use was protective (aOR = 0.36, 95% CI: 0.18–0.70), yet universal coverage was suboptimal: only 54.5% of households met coverage criteria despite 81.8% ownership, indicating gaps due to insufficient nets, wear-and-tear, improper use, or alternative sleeping arrangements^17^ .

It is important to note that the baseline survey did not include variables capturing “housing modification.” The “improved housing’’ indicator reflected general construction features (e.g., plastered or brick walls, cement floors, intact ceilings, iron roofs) rather than mosquito-entry barriers. Many such houses still had open eaves or unscreened windows, so a lack of association with infection risk is unsurprising. Accordingly, this finding does not preclude the potential impact of targeted structural interventions such as the 3D-WDS.

*Anopheles funestus* s.l. accounted for 70.2% of indoor CDC light trap collections, compared with 29.8% for *An. gambiae* s.l. Despite this predominance, malaria prevalence correlated significantly with *An. gambiae* s.l. (*r* = 0.5274, *p* = 0.0169) but not with *An. funestus* s.l. (*p* = 0.0635). The absence of a statistically significant association for *An. funestus* s.l., despite its numerical dominance, likely reflects the limited number of clusters (*n* = 20) and relatively homogeneous vector densities across sites, reducing statistical power. Nevertheless, *An. funestus* s.l. is recognized as one of the most efficient malaria vectors in East Africa because of its long lifespan, endophilic behaviour, and high anthropophily, and thus remains epidemiologically important^18–20^ .

Blood-meal analysis indicated higher human blood indices for *An. funestus s.s.* (HBI = 0.64) than for *An. gambiae s.s.* (HBI = 0.32), confirming both as competent, anthropophilic vectors. In our study, *An. funestus s.s.* was more abundant than *An. gambiae s.s.* in indoor collections, consistent with its well-known endophilic behaviour. This higher indoor density increases its likelihood of encountering human hosts and may partly explain the elevated HBI observed for this species.

The WHO cylinder bioassay results confirm substantial pyrethroid resistance in the local *Anopheles gambiae s.l.* population, consistent with recent resistance trend analyses from mainland Tanzania^21^ and comparable findings across East Africa, including western Kenya [22], where reduced pyrethroid efficacy has been widely documented. This regional pattern raises concerns about the operational performance of pyrethroid-based interventions^23,24^. Declining mosquito mortality directly undermines the community protection provided by ITNs and IRS.

The phenotypic resistance observed is supported by the presence of the kdr-East (L1014S) mutation, a well-established marker of target-site insensitivity^25^, and is likely further reinforced by metabolic mechanisms, particularly cytochrome P450–mediated detoxification, which are widespread among East African vector populations^26^ .

The kdr-East allele frequency differed markedly between sibling species: 45.0% in *Anopheles gambiae s.s.* compared with only 1.4% in *Anopheles arabiensis*. This disparity reflects species-specific bionomics and the resulting differences in exposure to insecticide selection pressure. The strongly anthropophilic and endophilic behavior of *An. gambiae s.s.* results in frequent contact with pyrethroids on ITNs and IRS-treated surfaces, thereby promoting selection for kdr. In contrast, the more exophilic, exophagic, and zoophilic tendencies of *An. arabiensis* reduce its exposure to indoor insecticides, explaining its very low kdr frequency^27^. These contrasting resistance profiles highlight the adaptive challenges posed by vector species contributing to residual, outdoor-focused transmission.

This study has limitations. The cross-sectional design provides only a single-season snapshot rather than longitudinal trends. Although target-site resistance (kdr) and phenotypic resistance were characterized, metabolic resistance mechanisms were not assessed, despite being recognized as major contributors to pyrethroid resistance in both *An. gambiae* s.l. and *An. funestus* s.l. Incorporating synergist bioassays or transcriptomic and proteomic profiling in future studies would strengthen inference regarding the underlying drivers of resistance.

Despite these challenges, the 3D-WDS offers a promising non-insecticidal complement to long-lasting insecticidal nets (LLINs) and indoor residual spraying (IRS) by physically blocking mosquito entry and trapping host-seeking mosquitoes between double screens. By reducing human–vector contact without imposing additional selection pressure for insecticide resistance, the 3D-WDS represents a context-appropriate intervention for communities facing heterogeneous vector species composition and high levels of pyrethroid resistance.

## Methods

### Study area

This study was conducted in 20 hamlets across 17 villages in Muheza District, Tanga Region, northeastern Tanzania (Fig. 1). Muheza lies at the foothills of the East Usambara Mountains and approximately 30 km from the Indian Ocean, an area characterized by a humid tropical climate with annual rainfall of 1,000–2,000 mm. Rainfall is bimodal, with short rains from October to December and long rains from March to June, producing two seasonal peaks in malaria transmission.

Most households are constructed from mud or cement with thatched or iron roofs, structures that favour indoor resting by malaria vectors. Livelihoods are predominantly small-scale farming, creating numerous temporary aquatic habitats during the rainy seasons^28^.

Malaria transmission in Muheza is driven primarily by *Anopheles gambiae s.l.* (mainly *An. gambiae s.s.* and *An. arabiensis*) and *Anopheles funestus s.l.*, which are abundant during the rainy seasons ; *An. funestus* s.l. remains common into the dry season and *Culex quinquefasciatus* is also widespread^29,30^. Previous studies have documented substantial pyrethroid resistance in *An. gambiae s.s.* populations in this area, including permethrin, deltamethrin and lambdacyhalothrin resistance^31^, and elevated frequencies of the CYP6P9a-R metabolic resistance allele in *An. funestus* s.l^32^.

### Site selection

Satellite imagery of Muheza district was obtained from Google Maps (Google LLC, https://www.google.com/maps), and OpenStreetMap (https://www.openstreetmap.org) was used to approximate the number of houses. After identifying several suitable localities with an average of 60–65 houses and considering factors like road accessibility and proximity to the project office, each community underwent an on-site visit and assessment to confirm their suitability. Subsequently, a total of 20 communities (clusters hereafter) were selected for the baseline survey.

### Community engagement

Community consent to participate in the cross-sectional study was obtained through community sensitization. A series of meetings were organized with village leaders, elders, residents of respective study clusters as well as community health workers and district medical officers of Muheza district. Letters of introduction and invitation were dispatched to the district medical office of Muheza and respective village leaders, sub-leaders, and hamlet leader. During the meetings, the intervention and study objectives were explained, along with the benefits for the participants as well as the community. The sessions included discussions on entomological and clinical procedures and other practical considerations. Each meeting concluded with a question-and-answer session, where participants’ questions were addressed. All interactions were conducted in *Kiswahili*. In addition to community consent, verbal and written consent were obtained at the household level from the head of the household, caregivers, or guardians of children in the respective households.

### Socio-demographic survey

Socio-demographic survey initiated with household identification and enumeration. The survey team was provided with a pre-generated unique numerical sequence to label identified households. Only residential structures were assigned these unique numbers, while buildings used for communal activities like community halls, school areas, and religious structures were not included in the enumeration process. Upon obtaining informed consent at the household level, participants were asked to provide basic information on household and identify the household head. Information regarding family size, housing condition, economic engagements, access to and use of bed nets and, malaria prevention practices at the household level were solicited from household head or an adult member if the household head was unavailable. In addition, type of housing, GPS coordinates, number of sleeping spaces and the presence of children within the household, along with other relevant information were gathered during demographic studies. Data were collected using digital forms implemented in Open Data Kit (ODK, https://opendatakit.org) on Android devices and synchronized to a central database.

### Clinical data collection

The malariometric survey was conducted across all 20 clusters in June and July 2019. The primary aim of this cross-sectional survey was to determine the prevalence of malaria and anaemia among children aged 6 months to 14 years in the community. The survey began with the formation of a clinical screening team, including a study supervisor, sociologist, clinical officer, nurses, laboratory technicians, and a data entry personnel. The screening process started with the sociologist documenting consent and assent forms from caretakers and children over 8 years, respectively. Anthropometric measurements, including height, weight, and mid-upper arm circumference (MUAC) for children under 5, were taken, followed by the measurement of axillary temperature.

*Plasmodium* infection was assessed using a malaria Rapid Diagnostic Test (mRDT) (CareStart RDTs; HRP2/pLDH, (Pf/PAN), Diasys, Wokingham, UK), and anaemia was assessed by measuring haemoglobin concentration with a portable β-haemoglobin HemoCue^®^ Hb 201 + photometer (Hemocue^®^, Ängelholm, Sweden). Haemoglobin levels were classified based on age-specific thresholds established by the WHO^33^. Blood samples were then collected from all consenting and participating children using the Dried Blood Spot (DBS) technique on filter paper, following WHO guidelines^34^. A strip of grade 3 Whatman filter paper (15 cm x 2.54 cm) was used, with blood samples collected on one half, dried, and folded. Each DBS card was labelled with a sticker containing participant information, numbered, and placed in a zip bag with a silica gel pack to prevent moisture contamination.

Children who tested positive for malaria were treated with Artemether-Lumefantrine (ALu) (Coartem^®^, Novartis Pharma, Basel, Switzerland) as per the national malaria treatment guidelines established by the Tanzanian Ministry of Health. Clinical officers and study nurses supervised the procedure, providing instructions to caregivers on the proper administration of ALu tablets, emphasizing the importance of taking them with food and following the prescribed schedule. During the survey period, the clinical team also treated non-malaria illnesses, providing paracetamol for axillary temperatures ≥ 37.5 °C, amoxicillin for non-severe pneumonia, oral rehydration salts (ORS) plus zinc for gastroenteritis, and ferrous sulphate plus albendazole for anaemia (haemoglobin < 8 g/dl). Children under 2 years old and those with severe illnesses were referred to Teule District Hospital in Muheza town for further medical attention.

Results were recorded on paper forms during the screening, double-entered into a digital database using the ODK application and checked for consistency. Erroneous data were verified and corrected.

### Adult mosquito collection

Adult mosquito collection was conducted using U.S. Centres for Disease Control and Prevention miniature light traps (CDC-LTs). Three houses were randomly selected from each cluster each night for 12 nights, yielding 36 trap-nights per cluster. This sampling intensity was determined based on logistical feasibility and the need to obtain representative cluster-level estimates of indoor mosquito density. In each selected household, a CDC-LT was hung indoors, 1 m above the floor and positioned approximately 50 cm away from the bed where a household member slept under a bed net. The traps were set at 18:00 and retrieved the next morning at 08:00. Each day, the trap and battery status were checked, and the traps were returned to the inventory for recharging and maintenance. The collected mosquito samples were retrieved the following morning and taken to the sorting facility, where they were identified to species by microscopy using standard morphological keys. The numbers of *An. gambiae* s.l., *An. funestus* s.l., and other Anophelines and Culicines captured were documented. Female Anophelines were sorted and stored for subsequent molecular analyses, while males were discarded.

### Insecticide susceptibility assays

Breeding sites within all 20 clusters were surveyed between May and August 2019. Mosquito larvae and pupae were collected from multiple breeding sites in the selected candidate villages and raised to adulthood in the insectary at NIMR, Amani. Adult *An. gambiae* s.l. identified morphologically were then separated from the cage and subjected to WHO’s cylinder test for insecticide susceptibility using following standard procedures^35^. Each test involved exposing four sets of 15–25 female *An. gambiae* s.l. per tube to WHO-recommended discriminating concentrations. 0.05% deltamethrin and 0.75% permethrin impregnated papers obtained from University Sains Malaysia, as well as control groups exposed to silicone oil impregnated paper. During the exposure phase, the knock-down (KD) rates were documented at intervals of 10, 15, 20, 30, 40, 50, and 60 min. A mosquito was considered knocked down if it was lying on its side on the floor of the exposure tube and incapable of flight. Following exposure, the mosquitoes were transferred to holding tubes lined with untreated paper, aided by gentle airflow between the tubes. A piece of cotton soaked in 10% sugar solution was placed on top of the holding tube. Mortality rates were recorded after a 24-hour holding period following exposure. The susceptibility status was evaluated based on the WHO criteria i.e., 98–100% mortality indicate susceptibility; 90–97% mortality required confirmation, and less than 90% mortality indicate resistance^35^. When control mortality between 5% and 20% was recorded, the mean observed mortality was corrected using Abbott’s formula^36^. In this study, control mortality did not exceed 10% in any assay; therefore, no test required repetition or exclusion according to WHO guidelines. The knockdown time for 50% (KDT_50_) and 95% (KDT_95_) of exposed mosquitoes were assessed using log-probit analysis^37^.

### Molecular analyses

#### Specimens for analyses and sample preparation

Female specimens of *An. gambiae* s.l. and *An. funestus* s.l. captured using CDC-LTs were morphologically identified, dissected into head and thorax, and genomic DNA was extracted separately from each part using 2% cetyltrimethylammonium bromide (CTAB), following the method outlined by Yahouedo et al.^20^. The DNA samples were labelled and stored at -20 °C for subsequent PCR and qPCR assays. The thorax was analyzed using a multiplex PCR to identify sibling species, while visibly engorged specimens were tested for blood meal sources through multiplex PCR. The head was examined for the presence of *Plasmodium* sporozoites using PCR. Additionally, a subset of approximately 50% of live and dead *An. gambiae* specimens from the WHO insecticide susceptibility tests were genotyped for sibling species identification and to detect the presence of the knockdown resistance-East (kdr-East) L1014S allele using quantitative real-time PCR (qPCR).

### Sibling species identification

Species identification of both *An. gambiae* s.l. and *An. funestus* s.l. was performed using multiplex PCR methods as described previously by Scott et al.^38^ and Koekemoer et al.^39^, respectively. For *An. gambiae* s.l., the multiplex PCR conditions included a single universal forward primer (UN; GTGTGCCCCTTCCTCGATGT) and four reverse primers for *An. gambiae* s.s. (GA; CTGGTTTGGTCGGCACGTTT), *An. arabiensis* (AR; AAGTGTCCTTCTCCATCCTA), *An. merus* (ME; TGACCAACCCACTCCCTTGA), and *An. quadriannulatus* (QD; CAGACCAAGATGGTTAGTAT). The thermal cycling conditions were as follows: an initial denaturation at 95 °C for 15 min, followed by 30 cycles of 94 °C for 30 s, 50 °C for 30 s, and 72 °C for 30 s, with a final extension at 72 °C for 10 min.

Similarly, for *An. funestus* s.l., the multiplex PCR conditions included a single universal forward primer (UV; TGTGAACTGCAGGACACAT) and five reverse primers for *An. funestus* s.s. (FUN; GCATCGATGGTTAATCATG), *An. rivulorum* (RIV; CAAGCCGTTCGACCTGATT), *An. parensis* (PAR; TGCGGTCCCAAGCTAGGTTC), *An. leesoni* (LEES; TACACGGGCGCCATGTAGTT), and *An. vaneedeni* (VAN; TGTCGACTTGGTAGCCGAAC). The thermal cycling conditions were an initial denaturation at 95 °C for 15 min, followed by 35 cycles of 94 °C for 30 s, 50 °C for 30 s, and 72 °C for 1 min, with a final extension at 72 °C for 10 min.

The master mix for both PCR reactions was prepared in a 20 µl (µl) solution containing 4 µl of HOT FIREPol^®^ MultiPlex Mix Ready to Load (Solis Biodyne, Tartu, Estonia), 25 pmol/µl of each primer, 2 ng/µl of genomic DNA, and molecular biology grade PCR water to adjust the final volume to 20 µl. Amplified fragments were run on a 1.5% agarose gel, visualized, and analysed on a portable mini-LED transilluminator (Invitrogen, USA).

### Knock down resistance (kdr) genotyping

*An. gambiae* s.l. specimens from WHO susceptibility tests were screened for L1014S kdr-East mutations using TaqMan q-PCR assay^40^. L1014S kdr-East mutation was screened using a forward primer kdr-F (5′-CAT TTT TCT TGG CCA CTG TAGTGA T-3′), and a reverse primer kdr-R (5′-CGA TCT TGG TCC ATGTTA ATT TGC A-3′), and probes WT (5′-CTT ACGACT AAA TTT C-3′) labelled with VIC™ at the 5′ end and kdr-E (5′-ACGACT GAA TTT C-3′) labelled with 6-FAM™ for the detection of the wild-type allele and the mutant L1014S allele respectively.

Each reaction included at least one non-template control (double de-ionized molecular grade water) and a known positive control for homozygous resistant, heterozygous, and homozygous susceptible allele. Both PCR reactions were prepared in a 20 µl of mixture consisting of 2.5 ng/ml of mosquito genomic DNA, 4 µl of 5x HOT FIREPol^®^ Multiplex qPCR Mix (Solis Biodyne, Tartu, Estonia), 800 nM of each primer and 200 nM of each probe. The cycling conditions were set at 95 °C for 10 min followed by 35 cycles of 95 °C for 15 s and 63 °C for 1 min. Allelic distribution from RT-qPCR assay was visualized and analysed on XY scatter plot ($\:\varDelta\:$R 6-FAM Vs $\:\varDelta\:$R VIC) using Aria Real-Time PCR Software version 1.8 (Agilent technologies, Santa Clara, USA, https://www.agilent.com). Allele frequencies were calculated directly from genotype counts [p=(2RR + RS)/(2 N)]. Associations between genotype and susceptibility (surviving vs. dead) were analysed using Pearson’s χ² test, or Fisher’s exact test when expected cell counts were < 5.

### Detection of *Plasmodium* in mosquito specimens

Malaria parasites in mosquito specimens collected from CDC-LTs were detected using a PCR method described previously^41^. The primer pair COX-IF (5′ AGAACGAACGCTTTTA ACGCCTG 3′) and COX-IR (3′ ACTTAATGGTGGATATAAAGTCCATCCwGT 5′) were used to amplify a 540 bp polymorphic fragment in the mitochondrial Cytochrome Oxidase I (COX-I) gene from *Plasmodium*. PCR reactions were prepared using 4 µl of 5x HOT FIREPol^®^ MultiPlex Mix Ready to Load master mix (Solis Biodyne, Tartu, Estonia), 5 µM of each primer (COX-IF and COX-IR), 2 µl of template DNA, and molecular biology grade water to bring the total reaction volume to 20 µl. Amplification was carried out with the following cycling parameters: 94 °C for 15 min, 40 cycles of 94 °C for 30 s, 65 °C for 1 min, and 72 °C for 1 min, followed by 72 °C for 10 min. Amplified PCR products were visualized on a 2% agarose gel and analysed on a portable mini-LED transilluminator (Invitrogen, USA).

### Blood meal analyses

Blood meal sources of engorged specimens from CDC-LTs were identified using a multiplex PCR method described by Kent^42^. The reaction was set up to amplify a segment of the cytochrome b gene as the target, using species-specific forward primers for human (Human741F; GGCTTACTTCTCTTCATTCTCTCCT), cow (Cow121F; CATCGGCACAAATTTAGTCG), pig (Pig573F; CCTCGCAGCCGTACATCTC), dog (Dog368F; GGAATTGTACTATTATTCGCAACCAT), and goat (Goat894F; CCTAATCTTAGTACTTGTACCCTTCCTC), along with a universal reverse primer (UNREV1025; GGTTGTCCTCCAATTCATGTTA). Each reaction contained 4 µl of HOT FIREPol^®^ MultiPlex Mix Ready to Load (Solis Biodyne, Tartu, Estonia), 5 µM of each primer, and 3 µl of DNA adjusted with PCR-grade water to a total volume of 20 µl.

PCR cycling conditions were as follows: initial denaturation at 95 °C for 15 min, followed by 40 cycles of denaturation at 95 °C for 1 min, annealing at 58 °C for 1 min, and extension at 72 °C for 1 min, with a final extension step at 72 °C for 10 min. Amplified PCR products were visualized on a 2% agarose gel, and band sizes were interpreted as follows: 334 bp (human), 453 bp (pig), 132 bp (goat or sheep), 680 bp (dog), and 561 bp (cow). Visualisation was performed using a portable mini-LED transilluminator (Invitrogen, USA).

### Statistical analyses

Descriptive analyses and graphical outputs were generated using GraphPad Prism version 9 (GraphPad Software, San Diego, CA, USA, https://www.graphpad.com). Continuous variables were summarized using the mean, median, interquartile range (IQR), and standard deviation (SD), while categorical variables were summarized using frequencies and percentages. All inferential statistical analyses were conducted using R version 4.3.3 (R Foundation for Statistical Computing, Vienna, Austria, https://www.r-project.org). Spatial data processing and map generation were performed using QGIS version 3.34, an open-source geographic information system. (https://qgis.org).

Malaria prevalence was calculated as the proportion of children who tested positive by mRDT among all examined. Hemoglobin concentration was summarized as the mean (g/dL) across clusters. Vector density and species composition were expressed as relative abundances based on the mean number and percentage of Anopheles mosquitoes collected per cluster using CDC light traps (CDC-LTs). The human biting rate (HBR) was defined as the number of Anopheles mosquitoes collected per person-night, and the entomological inoculation rate (EIR) was calculated as the product of HBR and the sporozoite rate. The human blood index (HBI) was calculated as the proportion of blood-fed mosquitoes with human-derived blood meals.

A household wealth index was constructed using principal component analysis (PCA), following the approach described by Vyas & Kumaranayake ^43^. Ten housing-related variables were included: wall type, roof type, window presence, window number, sleeping spaces, improved housing, income category, economic activity, cooking inside the house, and presence of curtains. These were converted into binary indicator variables and entered into PCA. The first principal component (PC1) was extracted as the wealth index and divided into quintiles (Q1 = poorest to Q5 = richest).

To examine risk factors for Plasmodium infection among children aged 6 months to 14 years, univariate logistic regression was first performed for each potential covariate. Variables with a p-value < 0.20 were considered eligible for multivariable modelling. A multilevel mixed-effects logistic regression model with a random intercept for cluster was used to account for the hierarchical study design and intra-cluster correlation. Backward elimination was applied to remove non-informative variables (*p* ≥ 0.20), while a priori covariates (child age group, child sex, household size, household head education, wealth quintile, and ITN access/use) were retained in all models regardless of significance. Adjusted odds ratios (aORs) with 95% confidence intervals (CIs) were reported. Model assumptions and multicollinearity were evaluated using residual diagnostics and variance inflation factors.

Cluster-level EIR was analysed using Negative Binomial regression, with the number of infected mosquitoes per cluster as the outcome, the log of trap-nights included as an offset, and cluster-level socio-demographic and housing characteristics included as fixed effects. This approach appropriately accounted for overdispersion and the aggregated nature of EIR data (Supplementary Table S2).

Species-specific EIR was evaluated using a species-by-cluster Negative Binomial model, also offset by trap-nights, with mosquito species (*An. gambiae s.l.* vs. *An. funestus s.l.*) included as a fixed effect alongside cluster-level covariates (Supplementary Table S4).

To compare *Plasmodium* infection probability between mosquito species at the individual level, a binomial Generalized Estimating Equation (GEE) with an exchangeable correlation structure and cluster as the grouping unit was fitted. Only mosquito species was included as a fixed effect to avoid pseudo-replication arising from repeating cluster-level covariates across individual mosquitoes. The GEE model provided robust standard errors accounting for non-independence of mosquitoes sampled within the same cluster. Results are presented in the main text.

Negative Binomial models were fitted using the MASS package, mixed-effects logistic regression using lme4, and the GEE model using geepack in R. Statistical significance was defined as *p* < 0.05 for all analyses.

### Ethical approval

This study was conducted in full compliance with the guidelines of the International Conference on Harmonization Tripartite Guideline for Good Clinical Practice (ICH-GCP), the Declaration of Helsinki, and the International Guidelines for Ethical Review of Epidemiological Studies. The study received ethical approval from the National Health Research Ethics Review Committee (NatREC), which includes the Medical Research Coordinating Committee (MRCC) and the Ministry of Health, Community Development, Gender, Elderly, and Children of the United Republic of Tanzania (certificate number NIMR/HQ/R.8c/Vol.I/1885), as well as from the Helsinki and Uusimaa Hospital District medical research ethics committee (reference number: 2242/2021). Before initiating the study, community consent was obtained both verbally and in writing from participating households. Village-level consent was obtained through community sensitization meetings with the broader objective of raising awareness about malaria and mosquito control. Community leaders of participating hamlets, district medical and public health officers, and health workers were invited to explain the clinical and entomological procedures. All questionnaires, surveys, and meetings were conducted in Kiswahili.

## Conclusions

Malaria burden in Muheza District remains substantial, with persistent transmission and high pyrethroid resistance. These findings support the urgent evaluation of non-insecticidal, house-based tools, such as the 3D-WDS, as complements to existing measures. This baseline provides the reference for the forthcoming cRCT and a foundation for optimising integrated, species-tailored vector control in similar endemic settings.

## Supplementary Information

Below is the link to the electronic supplementary material.

Supplementary Material 1

## Data Availability

All datasets supporting the conclusions of this study are provided within the article and its supplementary material (Additional file 1: Supplementary_materials.pdf).
