# Supplementary material for: Baseline malaria burden and pyrethroid resistance in Muheza, Tanzania informing a cluster randomized trial of the 3D window screens
Source: Sci Rep. 2026 Apr 3;16:15895. doi: 10.1038/s41598-026-46221-6 (PMC13194883; doi:10.1038/s41598-026-46221-6)
Supplement: Supplementary file 1 — Supplementary Material 1 [file 41598_2026_46221_MOESM1_ESM.pdf]

Supplementary materials

Table S1: Univariate logistic regression analysis of factors associated with *Plasmodium* infection among children aged 6 months–14 years

Variable	Number of children (N = 778)	Children with *Plasmodium* infection (%)	Odds Ratios (OR)	95% CI	p-value
HH head age group					
< Mean (Ref)	496	207 (41.7)	1		
> Mean	282	106 (37.5)	0.846	0.57 - 1.23	0.389
HH head gender					
Male (Ref)	560	222 (39.6)	1		
Female	218	91 (41.7)	1.191	0.81 - 1.74	0.372
HH head went to school?					
No (Ref)	35	15 (42.8)	1		
Yes	743	298 (40.1)	1.021	0.45 - 2.31	0.961
Economic activity					
Agriculture (Ref)	722	294 (40.7)	1		
Others	56	19 (33.9)	0.868	0.44 - 1.66	0.675
HH Size					
2 to 4 (Ref)	246	97 (39.4)	1		
5 to 7	403	161 (39.9)	1.038	0.68 - 1.56	0.859
> 7	129	55 (42.6)	1.085	0.57 - 2.04	0.801
Income					
Below average (Ref)	491	196 (39.9)	1		
Above average	287	117 (40.7)	1.181	0.79 - 1.76	0.413
Improved housing					
No (Ref)	539	228 (42.3)	1		
Yes	239	85 (35.5)	0.690	0.30 - 1.58	0.379
Number of sleeping spaces					
1 to 2 (Ref)	383	161 (42.0)	1		
3 to 4	361	138 (38.2)	0.888	0.58 - 1.34	0.580
> 5	34	14 (41.1)	0.925	0.35 - 2.32	0.869
ITN ownership					
No (Ref)	112	42 (37.5)	1		
Yes	666	271 (40.7)	0.87	0.58-1.32	0.524
Window present					
No (Ref)	10	4 (40.0)	1		
Yes	768	309 (40.2)	1.175	0.25 - 6.11	0.839
Number of windows					
< 3 (Ref)	236	102 (43.2)	1		
3 to 5	301	125 (41.5)	0.914	0.59 - 1.39	0.678
> 6	241	86 (35.6)	0.677	0.39 - 1.15	0.151
Curtain present					
No (Ref)	544	228 (41.9)	1		
Yes	234	85 (36.3)	0.907	0.62- 1.31	0.605

Eave type					
Closed (Ref)	78	24 (30.7)	1		
Open	700	289 (41.2)	1.592	0.86 - 2.99	0.140
Cooking inside the house					
No (Ref)	465	181 (38.9)	1		
Yes	313	132 (42.1)	1.097	0.76 - 1.57	0.615
Using additional mosquito control method					
No (Ref)	690	277 (40.1)	1		
Yes	88	36 (40.9)	1.042	0.60 - 1.77	0.880
Age group					
Below 5 (Ref)	314	86 (27.3)	1		
Above 5	464	227 (48.9)	3.072	2.16 - 4.39	<0.001
Gender (participated children)					
Male (Ref)	395	166 (42.0)	1		
Female	383	147 (38.3)	1.012	0.72 - 1.41	0.945
Slept under bed net last night?					
No (Ref)	61	37 (60.6)	1		
Yes	717	276 (38.5)	0.41	0.24 - 0.69	0.001
Wealth quintile					
Q1 (Poorest, Ref)	156	72 (46.15%)	1		
Q2	156	65 (41.66%)	0.95	0.61 – 1.48	0.824
Q3	156	58 (37.17%)	0.74	0.47 – 1.16	0.185
Q4	156	54 (34.61%)	0.69	0.45 – 1.08	0.106
Q5 (Richest)	154	44 (28.57%)	0.70	0.44 – 1.10	0.122

Table S2: Human biting rates

SN	Study Villages	*An. gambiae*				*An. funestus*				*Total Anophelines*			
		N	Mean (95% CI)	HBR (95% CI)	EIR	N	Mean (95% CI)	HBR (95% CI)	EIR	N	Mean (95% CI)	HBR (95% CI)	EIR
1	MBARAMO	20	1.67 (0.30-3.03)	0.55 (0.101-1.01)	0.00	22	1.83 (-0.29-3.96)	0.61 (-0.09-1.32)	0.00	42	3.5 (0.22-6.78)	1.17 (0.07-2.26)	0.00
2	KITUAMAWE	65	5.42 (-3.04-13.9)	1.81 (-1.01-4.63)	0.19	54	4.5 (0.36-8.63)	1.5 (0.12-2.88)	0.03	119	9.92 (-2.19-22)	3.31 (-0.72-7.34)	0.22
3	MTONGA	28	2.33 (0.89-3.78)	0.77 (0.29-1.26)	0.03	52	4.33 (0.30-8.36)	1.45 (0.10-2.79)	0.08	80	6.67 (1.69-11.6)	2.22 (0.56-3.88)	0.11
4	KIDUTANI	37	3.08 (0.97-5.19)	1.03 (0.32-1.73)	0.00	65	5.42 (1.71-9.13)	1.81 (0.56-3.04)	0.14	102	8.5 (4.5-12.5)	2.83 (1.5-4.17)	0.14
5	NGUGWINI	62	5.17 (1.91-8.43)	1.72 (0.63-2.81)	0.06	30	2.5 (-0.75-5.76)	0.833 (-0.25-1.92)	0.00	92	7.67 (2.48-12.9)	2.56 (0.82-4.28)	0.06
6	KWAJUMBE	136	11.3 (-5.68-28.3)	3.78 (-1.89-9.45)	0.11	85	7.08 (-2.81-17)	2.36 (-0.93-5.66)	0.00	221	18.4 (0.25-36.6)	6.14 (0.08-12.2)	0.11
7	MJIMPYA	11	0.91 (-0.11-1.95)	0.30 (-0.03-0.65)	0.00	61	5.08 (1.55-8.62)	1.7 (0.51-2.87)	0.00	72	6 (1.58-10.4)	2 (0.52-3.47)	0.00
8	JAMBE	27	2.25 (0.09-4.4)	0.75 (0.03-1.47)	0.06	28	2.33 (0.55-4.12)	0.77 (0.18-1.37)	0.11	55	4.58 (1.72-7.44)	1.53 (0.57-2.48)	0.17
9	POTWE	8	0.67 (0.10-1.23)	0.22 (0.03-0.41)	0.00	29	2.42 (0.38-4.45)	0.80 (0.12-1.48)	0.00	37	3.08 (1.03-5.14)	1.03 (0.34-1.71)	0.00
10	KIUMBA	14	1.17 (0.23-2.1)	0.38 (0.07-0.69)	0.03	89	7.42 (-4.8-19.7)	2.47 (-1.61-6.55)	0.00	103	8.58 (-4.08-21.2)	2.86 (-1.36-7.08)	0.03
11	KWASABIA	75	6.25 (-1.33-13.8)	2.08 (-0.44-4.61)	0.08	47	3.92 (0.02-7.81)	1.31 (0.01-2.6)	0.03	122	10.2 (1.03-19.3)	3.39 (0.34-6.43)	0.11
12	MKINGA	35	2.92 (-0.9-6.73)	0.97 (-0.3-2.24)	0.03	187	15.6 (0.76-30.4)	5.19 (0.25-10.1)	0.06	222	18.5 (-0.07-37.1)	6.17 (-0.02-12.4)	0.08
13	MIANZINI	37	3.08 (-0.98-7.15)	1.03 (-0.32-2.38)	0.00	254	21.2 (2.57-39.8)	7.06 (0.85-13.3)	0.00	291	24.3 (2.45-46)	8.08 (0.81-15.3)	0.00
14	MSEKO	22	1.83 (-0.17-3.84)	0.61 (-0.05-1.28)	0.03	82	6.83 (-0.63-14.3)	2.28 (-0.20-4.77)	0.03	104	8.67 (-0.33-17.7)	2.89 (-0.11-5.89)	0.06
15	MADAGO	16	1.33 (-0.13-2.8)	0.44 (-0.04-0.93)	0.00	49	4.08 (0.94-7.22)	1.36 (0.31-2.41)	0.00	65	5.42 (1.36-9.48)	1.81 (0.45-3.16)	0.00
16	KWEMWALE	15	1.25 (0.03-2.47)	0.41 (0.01-0.82)	0.03	163	13.6 (-2.72-29.9)	4.53 (-0.90-9.96)	0.08	178	14.8 (-1.92-31.6)	4.94 (-0.64-10.5)	0.11
17	MASIMBANI	25	2.08 (0.71-3.45)	0.69 (0.23-1.15)	0.00	192	16 (0.99-31)	5.33 (0.33-10.3)	0.11	217	18.1 (2.07-34.1)	6.03 (0.68-11.4)	0.11
18	KWAMNYESE	52	4.33 (-0.54-9.21)	1.45 (-0.18-3.07)	0.03	82	6.83 (-1.65-15.3)	2.28 (-0.55-5.1)	0.03	134	11.2 (-1.44-23.8)	3.72 (-0.48-7.92)	0.06
19	KIDAGE	24	2 (-0.45-4.45)	0.66 (-0.15-1.49)	0.00	89	7.42 (0.18-14.6)	2.47 (0.06-4.88)	0.03	113	9.42 (0.07-18.8)	3.14 (0.02-6.25)	0.03
20	KITOPENI	38	3.17 (0.61-5.72)	1.06 (0.20-1.91)	0.06	101	8.42 (1.37-15.5)	2.81 (0.45-5.15)	0.06	139	11.6 (2.62-20.5)	3.86 (0.87-6.85)	0.11

N represents the total number of female specimens collected using CDC-LTs throughout the study period; the mean indicates the average number of mosquitoes collected per village collection night; HBR stands for Human Biting Rate, and EIR refers to the Entomological Inoculation Rate.

Table S3: Negative Binomial regression model for cluster-level EIR.

Variable	Coefficient (log scale)	Standard Error	Rate Ratio (RR)	95% CI	p-value
const	-35.85	20.34	0.00	0.00 – 5.53×10^1	0.08
mean_HH_age	0.15	0.12	1.16	0.91 – 1.46	0.23
prop_male_HH	5.15	4.65	1.72×10^2	2×10^-2 – 1.56×10^6	0.27
prop_no_edu	0.40	7.26	1.50	0.00 – 2.28×10^6	0.96
mean_hh_size	1.51	0.93	4.51	0.73 – 2.78×10^1	0.10
mean_sleep_spaces	1.77	5.40	5.89	0.00 – 2.32×10^5	0.74
mean_nets	0.68	0.69	1.97	0.51 – 7.62	0.33
mean_windows	3.73	2.79	4.18×10^1	1.8×10^-1 – 9.88×10^3	0.18
prop_open_eaves	-5.07	6.00	1×10^-2	0.00 – 7.97×10^2	0.40
mean_wealth_index	-13.17	10.25	0.00	0.00 – 1.00×10^3	0.20

Model estimates for the association between household- and structural-level factors and cluster-level entomological inoculation rate (EIR). Rate ratios (RR) and 95% confidence intervals (CI) are shown. Some variables have extremely wide CIs due to low variability, sparse data, or near-zero counts in certain clusters, resulting in unstable estimates.

Table S4: Species-specific Negative Binomial regression model comparing *An. gambiae s.l.* and *An. funestus s.l.* EIR.

Variable	Coefficient (log scale)	Standard Error	Rate Ratio (RR)	95% CI	p-value
const	-35.54	16.65	0.00	0.00 – 5×10^-2	0.03
species_AF	-0.03	0.45	0.97	0.40 – 2.37	0.96
mean_HH_age	0.13	0.10	1.14	0.94 – 1.38	0.17
prop_male_HH	4.70	3.91	1.10×10^2	5×10^-2 – 2.33×10^5	0.23
prop_no_edu	0.83	5.91	2.30	0.00 – 2.46×10^5	0.89
mean_hh_size	1.39	0.77	4.01	0.89 – 1.80×10^1	0.07
mean_sleep_spaces	2.08	4.43	7.99	0.00 – 4.72×10^4	0.64
mean_nets	0.60	0.57	1.82	0.60 – 5.54	0.29
mean_windows	3.72	2.30	4.15×10^1	4.6×10^-1 – 3.73×10^3	0.10
prop_open_eaves	-5.23	4.80	1×10^-2	0.00 – 6.50×10^1	0.28
mean_wealth_index	-13.17	8.42	0.00	0.00 – 2.82×10^1	0.12

Rate ratios (RR) and 95% confidence intervals (CI) from a negative binomial model assessing associations between household and structural factors and cluster-level EIR. Some variables have extremely wide CIs due to low variability, sparse data, or near-zero counts in certain clusters, resulting in unstable estimates.

Table S5: Blood meal sources and host seeking behaviour of Anophelines

Species	No. analysed N (HBI)	Blood meal Sources						
		Human N (HBI)	Bovine N (BBI)	Human + Bovine Mixed N (BMI)	Dog N (BMI)	Goat N (BMI)	Human + Goat Mixed N (BMI)	Unknown N (BMI)
An. gambiae								
An. gambiae s.s.	59 (0.32)	17 (0.28)	1 (0.01)	0 (0.00)	0 (0.00)	0 (0.03)	2 (0.03)	39 (0.66)
An. arabiensis	11 (0.36)	3 (0.27)	0 (0.00)	0 (0.00)	0 (0.00)	1 (0.18)	1 (0.09)	6 (0.54)
Total *An. gambiae*	70 (0.32)	20 (0.28)	1 (0.01)	0 (0.00)	0 (0.00)	1 (0.05)	3 (0.04)	45 (0.64)
An. funestus								
An. funestus s.s.	157 (0.68)	106 (0.67)	7 (0.05)	1 (0.006)	4 (0.025)	4 (0.02)	0 (0.00)	35 (0.22)
An. leesoni	2 (1.00)	2 (1.00)	0 (0.00)	0 (0.00)	0 (0.00)	0 (0.00)	0 (0.00)	0 (0.00)
An. rivulorum	14 (0.14)	2 (0.14)	4 (0.28)	0 (0.00)	1 (0.07)	2 (0.14)	0 (0.00)	5 (0.35)
Total *An. funestus*	173 (0.64)	110 (0.63)	11 (0.07)	1 (0.005)	5 (0.028)	6 (0.03)	0 (0.00)	40 (0.23)
Overall *Anopheles*	243 (0.55)	130 (0.53)	12 (0.05)	1 (0.004)	5 (0.02)	7 (0.04)	3 (0.01)	85 (0.34)

HBI = Human Blood Meal Index, BBI = Bovine Blood Meal Index, BMI = Blood Meal Index.

When calculating the human blood index (HBI), bovine blood index (BBI), and other blood meal index (BMI), mixed blood meals were included in the counts for human, bovine, and goat blood meals. Samples with no visible band for cytochrome b gene fragment during visualization were classified as unknown blood meals and represents the overall BMI.

Table S6: Susceptibility of *An. gambiae s.l.* to the diagnostic dose of common pyrethroids: Permethrin and Deltamethrin

		Permethrin 0.75%						Deltamethrin 0.05%					
SN	Cluster	N	Alive	Dead	kd50 (95% CI)	kd95 (95% CI)	CoM (%)	N	Alive	Dead	kd50 (95% CI)	kd95 (95% CI)	CoM (%)
1	Mbaramo	60	23	37	36.41 (34.26–38.64)	59.15 (55.29–64.20)	61.7	60	23	37	36.41 (34.26–38.64)	59.15 (55.29–64.20)	61.7
2	Kituamawe	60	25	35	42.96 (37.75–49.01)	66.04 (57.74–82.61)	58.3	60	35	25	39.81 (36.09–44.18)	64.79 (58.04–75.65)	41.7
3	Mtonga	60	26	34	45.84 (43.71–48.13)	65.96 (62.06–71.29)	56.7	60	34	26	48.87 (45.53–54.11)	75.63 (66.96–91.17)	43.3
4	Kidutani	40	5	35	33.38 (30.55–36.35)	59.35 (54.16–66.63)	87.5	66	34	32	51.04 (47.12–56.22)	81.26 (72.68–95.12)	48.5
5	Ngugwini	80	47	33	45.63 (42.21–49.73)	74.49 (67.38–85.30)	41.3	60	35	25	39.09 (35.20–43.38)	62.94 (56.36–73.61)	41.7
6	Kwajumbe	80	66	14	36.13 (30.42–42.41)	61.39 (52.63–78.74)	17.5	80	47	33	60.26 (52.85–74.16)	100.93 (83.71–138.47)	41.3
7	Mjimpya	60	32	28	45.43 (42.68–48.60)	74.71 (68.80–82.90)	46.7	40	12	28	35.41 (30.74–40.48)	61.84 (54.07–75.42)	70.0
8	Jambe	40	17	23	36.89 (32.46–41.72)	59.20 (52.28–71.31)	57.5	40	3	37	25.29 (22.65–27.89)	47.94 (43.51–54.28)	92.5
9	Potwe	40	16	24	38.87 (34.87–43.32)	64.52 (57.50–75.76)	60.0	60	23	37	37.93 (33.96–42.33)	65.11 (57.92–76.73)	61.7
10	Kiumba	45	15	30	39.62 (36.39–43.28)	72.62 (65.59–82.88)	66.7	88	63	25	43.29 (37.06–51.83)	74.89 (62.88–101.21)	28.4
11	Kwasabia	40	26	14	36.67 (31.17–42.62)	57.38 (49.77–72.46)	35.0	47	1	46	27.43 (23.29–31.79)	46.79 (40.64–57.93)	97.9
12	Mkinga	100	77	23	47.21 (45.38–49.21)	70.11 (66.42–74.84)	23.0	100	68	32	44.43 (39.21–51.33)	76.12 (65.48–96.46)	32.0
13	Mianzini	60	30	30	44.11 (37.91–52.73)	73.02 (61.62–98.06)	50.0	54	19	35	36.13 (33.46–38.97)	61.41 (55.31–70.66)	64.8
14	Mseko	40	11	29	34.28 (29.18–39.76)	66.06 (56.90–82.69)	72.5	40	17	23	41.80 (37.11–47.57)	74.97 (66.19–92.16)	57.5
15	Madago	60	26	34	39.90 (35.94–44.41)	68.38 (60.85–80.51)	56.7	80	72	8	67.14 (61.80–75.38)	102.81 (90.88–122.52)	10.0
16	Kwemwale	60	23	37	36.67 (34.50–38.93)	59.64 (55.75–64.71)	61.7	60	24	36	36.89 (30.36–44.49)	67.09 (56.14–91.13)	60.0
17	Masimbani	69	13	56	35.64 (33.61–37.76)	59.13 (55.42–63.02)	81.2	40	26	14	22.08 (21.33–27.88)	32.77 (28.18–37.66)	35.0
18	Kwamnyese	78	42	36	49.01 (45.08–54.08)	82.11 (73.26–96.11)	45.6	40	13	27	44.65 (38.90–52.51)	77.59 (65.77–101.56)	67.5
19	Kidage	45	8	37	50.29 (47.52–53.60)	72.41 (66.86–80.82)	81.7	80	64	16	58.48 (52.45–68.54)	94.76 (80.94–121.69)	20.0
20	Kitopeni	40	12	28	40.56 (34.79–46.79)	60.45 (52.71–76.90)	68.8	80	16	64	47.92 (45.55–50.64)	75.22 (70.15–82.00)	80.0

N, represents the total number of adult mosquitoes exposed to permethrin and deltamethrin at diagnostic concentrations; CoM, denotes the corrected observed mortality after Abbott’s correction, expressed as a percentage.

Table S7: Frequencies of the kdr 1014S (kdr East) in *An. gambiae* species complex in study clusters.

SN	Cluster	Total						An. gambiae						An. arabiensis					
		N	Genotype			R (freq)	95% CI	N	Genotype			R (freq)	95% CI	N	Genotype			R (freq)	95% CI
			RR	RS	SS				RR	RS	SS				RR	RS	SS		
1	MBARAMO	58	2	1	55	0.04	(0.02–0.09)	2	1	1	0	0.75	(0.30–0.95)	56	1	0	55	0.02	(0.00–0.10)
2	KITUAMAWE	58	5	3	50	0.11	(0.06–0.18)	7	5	2	0	0.86	(0.49–0.97)	51	0	1	50	0.01	(0.00–0.05)
3	MTONGA	60	5	1	54	0.09	(0.05–0.16)	4	3	1	0	0.88	(0.47–0.99)	56	2	0	54	0.04	(0.01–0.09)
4	KIDUTANI	54	1	2	51	0.04	(0.01–0.09)	3	1	2	0	0.67	(0.21–0.94)	51	0	0	51	0.00	(0.00–0.07)
5	NGUGWINI	68	3	0	65	0.04	(0.02–0.09)	12	0	0	12	0.00	(0.00–0.25)	56	2	0	54	0.04	(0.01–0.09)
6	KWAJUMBE	79	11	3	65	0.16	(0.10–0.23)	11	9	2	0	0.91	(0.63–0.98)	68	2	1	65	0.04	(0.01–0.08)
7	MJIMPYA	50	2	2	46	0.06	(0.02–0.13)	11	1	1	9	0.14	(0.04–0.39)	39	1	1	37	0.04	(0.01–0.12)
8	JAMBE	40	1	0	39	0.03	(0.00–0.09)	1	1	0	0	1.00	(0.51–1.00)	39	0	0	39	0.00	(0.00–0.09)
9	POTWE	49	2	0	47	0.04	(0.01–0.10)	2	0	0	2	0.00	(0.00–0.51)	47	2	0	45	0.04	(0.01–0.10)
10	KIUMBA	66	4	1	61	0.07	(0.03–0.13)	11	4	1	6	0.41	(0.18–0.69)	55	0	0	55	0.00	(0.00–0.06)
11	KWASABIA	44	4	3	37	0.13	(0.06–0.22)	21	4	3	14	0.26	(0.14–0.45)	23	0	0	23	0.00	(0.00–0.12)
12	MKINGA	99	8	2	89	0.09	(0.06–0.14)	11	7	2	2	0.73	(0.43–0.91)	88	1	0	87	0.01	(0.00–0.04)
13	MIANZINI	56	0	1	55	0.01	(0.00–0.05)	0	0	0	0	NA	NA	56	0	1	55	0.01	(0.00–0.05)
14	MSEKO	40	0	0	40	0.00	(0.00–0.05)	1	0	0	1	0.00	(0.00–0.49)	39	0	0	39	0.00	(0.00–0.09)
15	MADAGO	70	0	1	69	0.01	(0.00–0.04)	7	0	1	6	0.07	(0.01–0.32)	63	0	0	63	0.00	(0.00–0.06)
16	KWEMWALE	59	3	1	55	0.06	(0.02–0.12)	4	3	1	0	0.88	(0.47–0.99)	55	0	0	55	0.00	(0.00–0.06)
17	MASIMBANI	55	2	1	52	0.05	(0.02–0.10)	4	2	1	1	0.63	(0.31–0.87)	51	0	0	51	0.00	(0.00–0.07)
18	KWAMNYESE	58	2	2	54	0.05	(0.02–0.11)	16	1	1	14	0.09	(0.03–0.26)	42	1	1	40	0.04	(0.01–0.13)
19	KIDAGE	62	9	3	50	0.17	(0.11–0.25)	11	9	2	0	0.91	(0.63–0.98)	51	0	1	50	0.01	(0.00–0.05)
20	KITOPENI	60	0	0	60	0.00	(0.00–0.03)	0	0	0	0	NA	NA	60	0	0	60	0.00	(0.00–0.06)
Total		1185	64	27	1094			139	52	21	66			1046	12	6	1028		

N, number of mosquitoes successfully genotyped; SS, homozygous susceptible; RS, heterozygous; RR, homozygous resistant. R (freq), resistant allele frequency.
